# Supplementary material for: Distributed control of motor circuits for backward walking in Drosophila
Source: Nat Commun. 2020 Dec 2;11:6166. doi: 10.1038/s41467-020-19936-x (PMC7710706; doi:10.1038/s41467-020-19936-x)
Supplement: Supplementary file 1 — Supplementary Information [file 41467_2020_19936_MOESM1_ESM.pdf]

Distributed control of motor circuits for backward walking in *Drosophila*

Kai Feng, Rajyashree Sen, Ryo Minegishi, Michael Dübbert, Till Bockemühl, Ansgar Büschges, and Barry J. Dickson

Supplementary Information

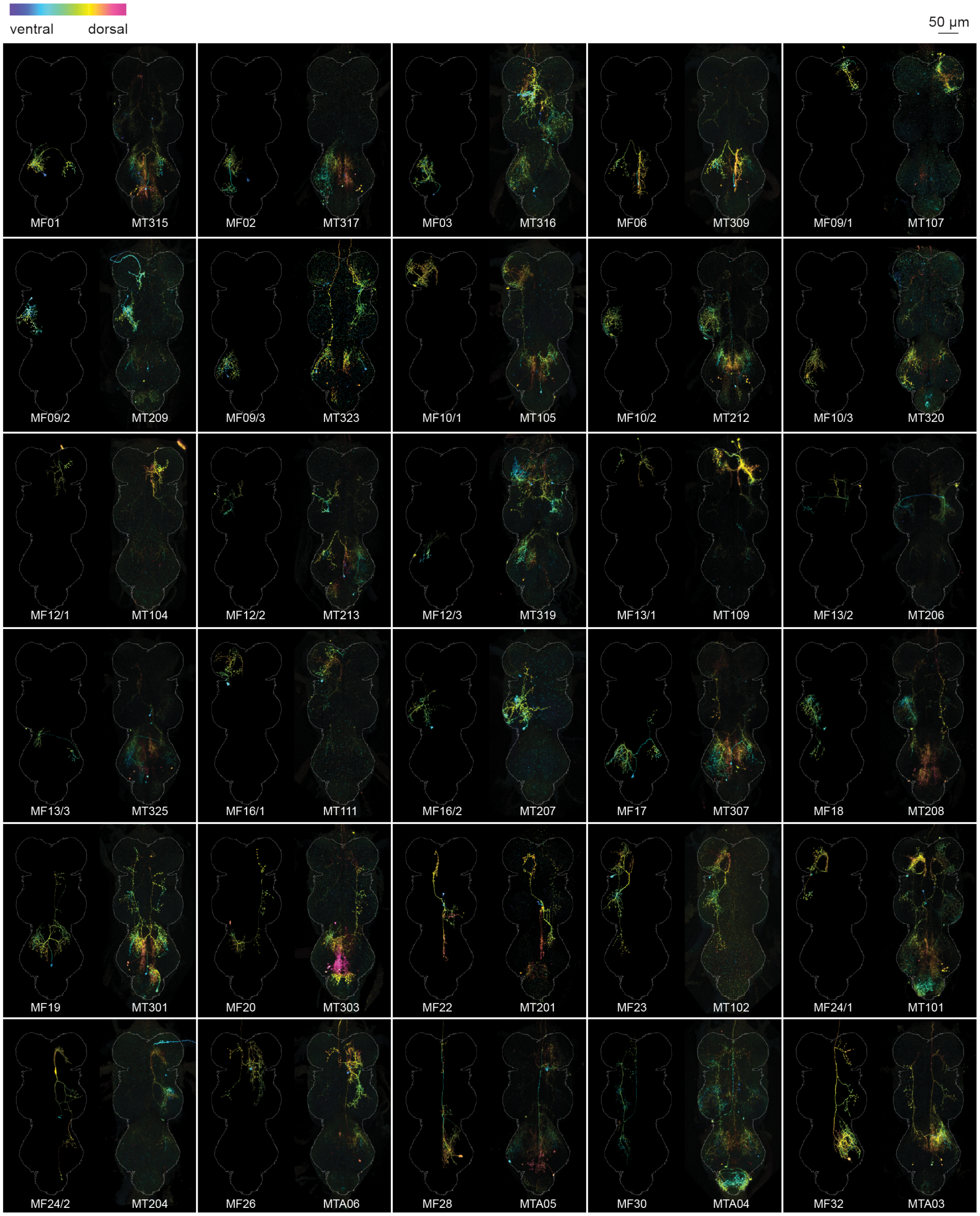

Supplementary Fig. 1. Comparison of cell types identified by trans-Tango and functional imaging. Cell types identified by both trans-Tango and functional imaging. All images are colorMIPs of registered confocal images. For each pair, the left image is an image of an MDN-responsive cell, segmented from an MCFO image obtained using the corresponding GAL4 driver. The right images is an unsegmented stochastic trans-Tango sample that appears to include the same cell type.

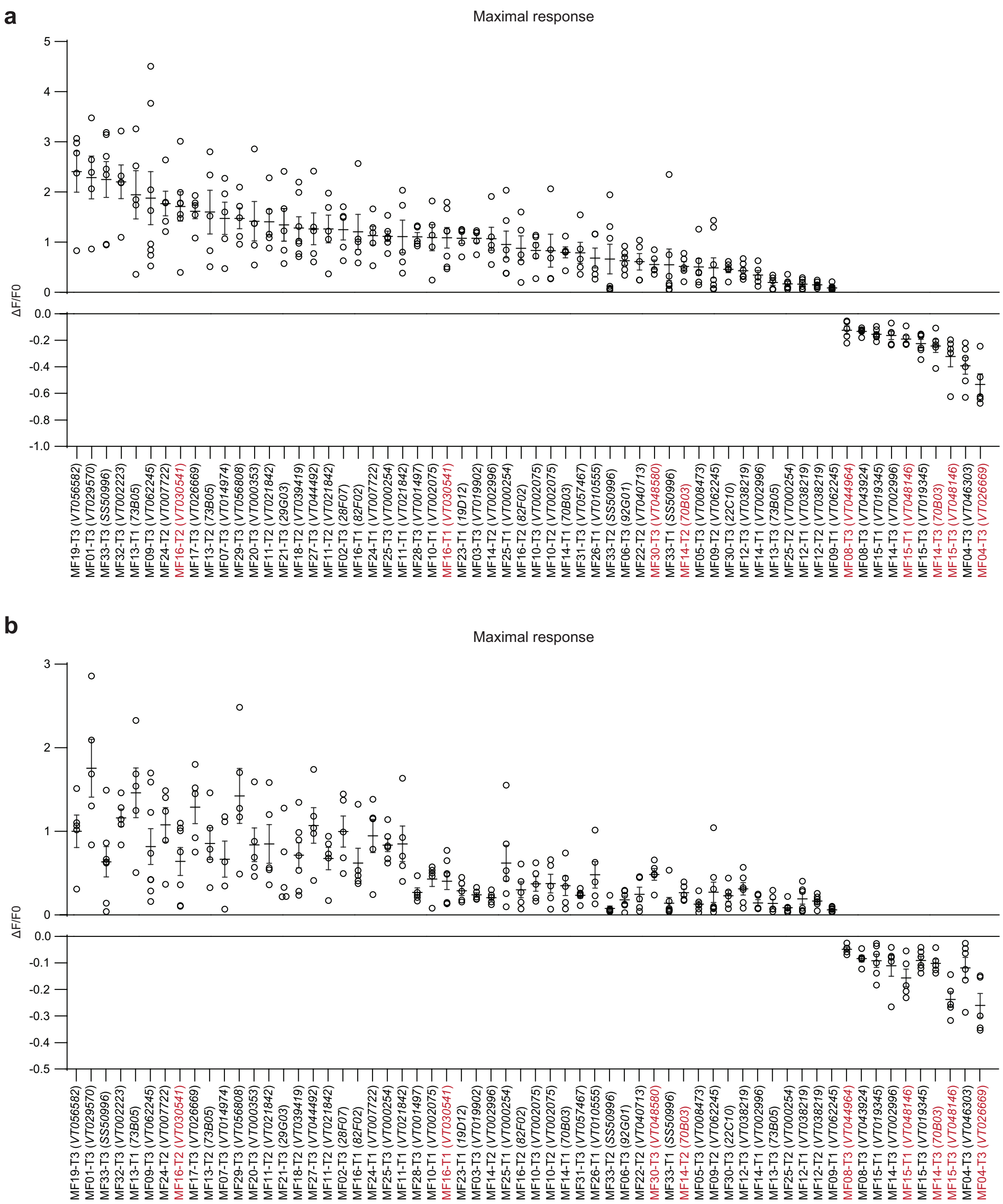

Supplementary Fig. 2. Calcium responses of MF cell types. Maximal ΔF/F responses of each cell upon (**a**) 20-s or (**b**) 1-s photoactivation of MDNs. Each data point (circle) represents a single fly, and bars show mean ± s.e.m. *N* ≥ 5 flies for each genotype. Samples are ordered in both plots by their response to the 20-s stimulus. "-T1", "-T2", and "-T3" suffixes indicate T1, T2, and T3 cells, respectively. For some cell types, two independent driver lines were used. Black font indicates SS lines shown in Fig. 3. Source data are provided as a Source Data file.

a

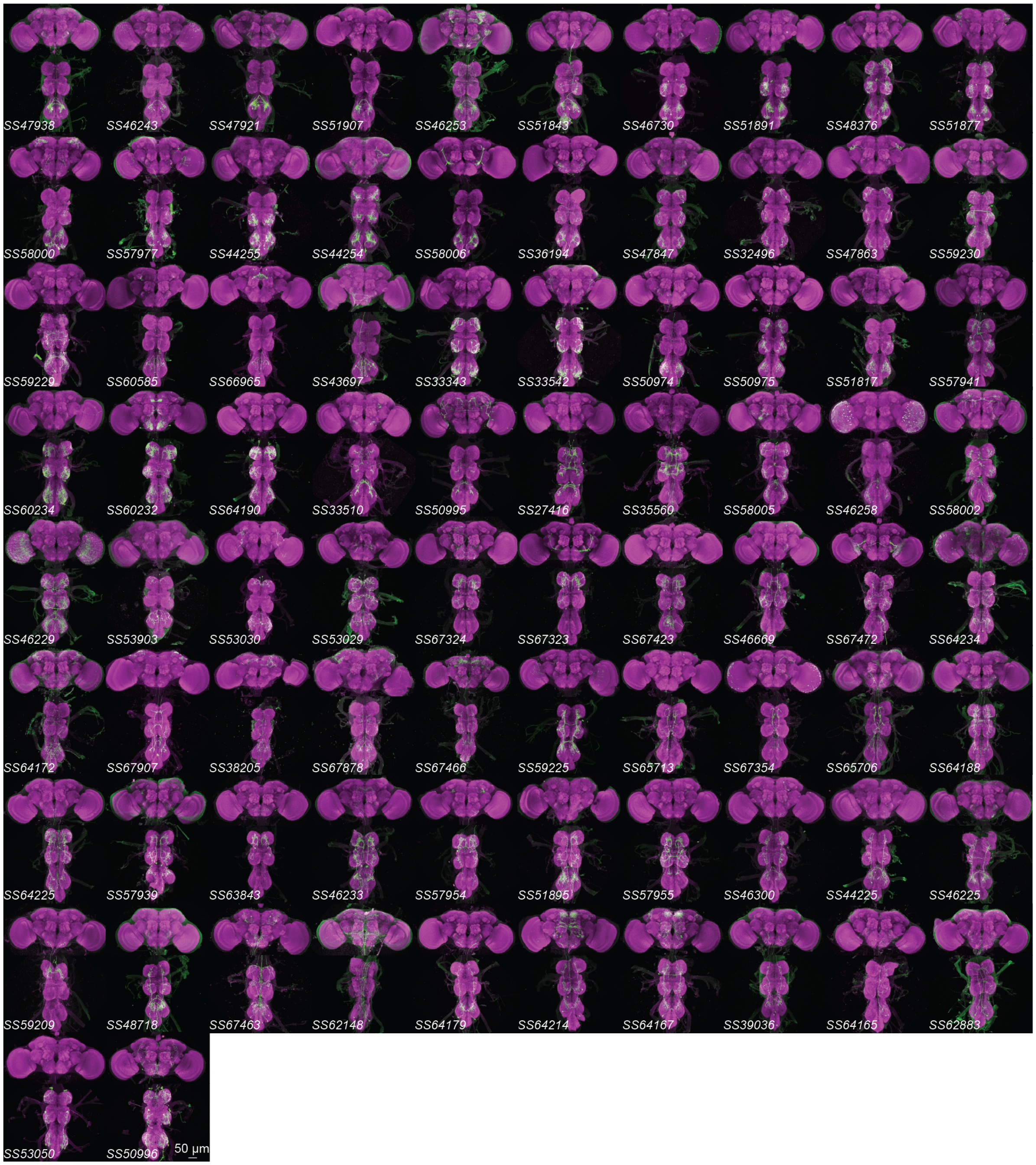
SS47938 SS46243 SS47921 SS51907 SS46253 SS51843 SS46730 SS51891 SS48376 SS51877
SS58000 SS57977 SS44255 SS44254 SS58006 SS36194 SS47847 SS32496 SS47863 SS59230
SS59229 SS60585 SS66965 SS43697 SS33343 SS33542 SS50974 SS50975 SS51817 SS57941
SS60234 SS60232 SS64190 SS33510 SS50995 SS27416 SS35560 SS58005 SS46258 SS58002
SS46229 SS53903 SS53030 SS53029 SS67324 SS67323 SS67423 SS46669 SS67472 SS64234
SS64172 SS67907 SS38205 SS67878 SS67466 SS59225 SS65713 SS67354 SS65706 SS64188
SS64225 SS57939 SS63843 SS46233 SS57954 SS51895 SS57955 SS46300 SS44225 SS46225
SS59209 SS48718 SS67463 SS62148 SS64179 SS64214 SS64167 SS39036 SS64165 SS62883
SS53050 SS50996
50 µm

b

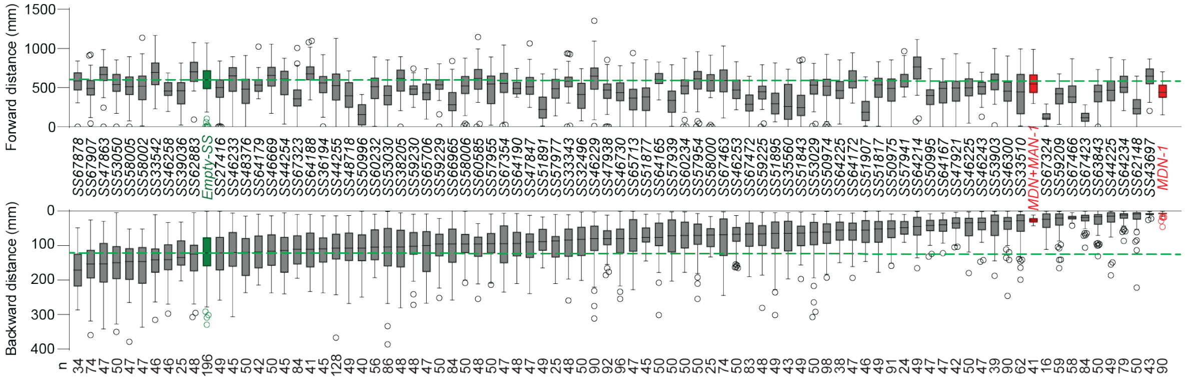
Forward distance (mm)
Backward distance (mm)
Empty-SS
MDN+MAN-1
MDN-1
n

Supplementary Fig. 3. Anatomical and functional analysis of split-Gal4 lines labeling MDN-downstream neurons. a, Confocal images of central nervous systems for each split-GAL4 line shown in Fig. 4a, stained to reveal all synapses (nc82, magenta) and the targeted neurons (CsChrimson-mVenus, detected using anti-GFP, green). **b**, Box-and-whisker plots for the forward walking distance during 45 s without optogenetic stimulation (top), and total backward walking distance for the same flies during 9 successive 5-s red light pulses. Boxes show median and interquartile ranges. Bars extend down to the larger of the minimum value or the 25 percentile minus 1.5 times the interquartile range, and up to the smaller of the maximum value or the 75 percentile plus 1.5 times the interquartile range. Outliers are shown as circles. Positive and negative control lines are labeled in red and green, respectively. Green dashed lines show the median for the negative control, for comparison. Lines are ordered by the median backward walking distance. Source data are provided as a Source Data file.

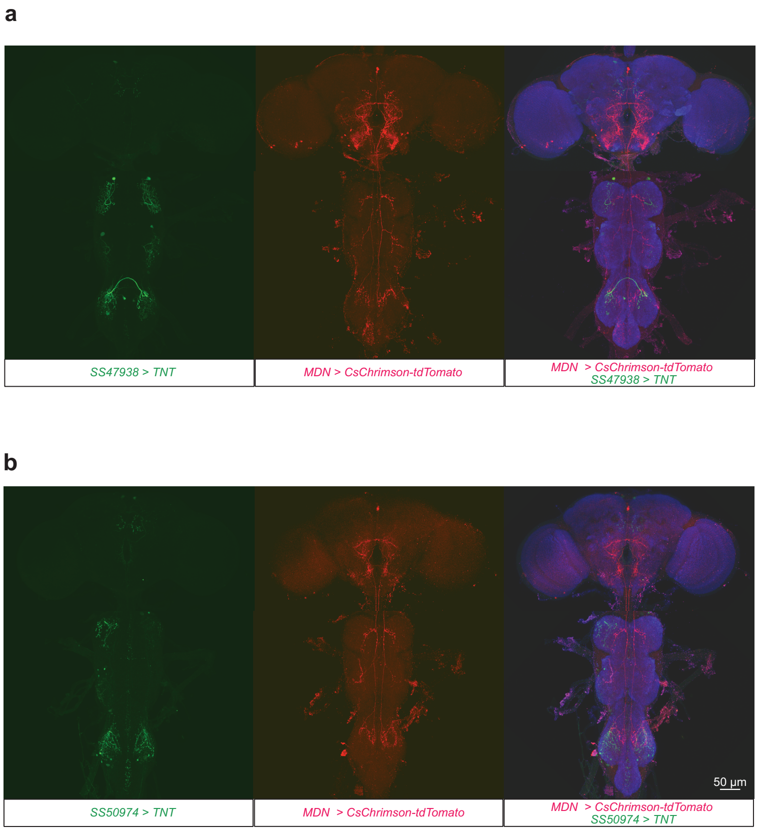

Supplementary Fig. 4. Expression of CsChrimson and TNT in flies with two split drivers. Confocal images of the central nervous systems of flies in which CsChrimson was expressed in MDN using a split-LexA driver and TNT was expressed in either LBL40 (**a**) or LUL130 (**b**) using a split-GAL4 driver. Samples were stained with anti-TNT (green), anti-RFP (to visualize CsChrimson-tdTomato, red) and nc82 (all synapses, blue).

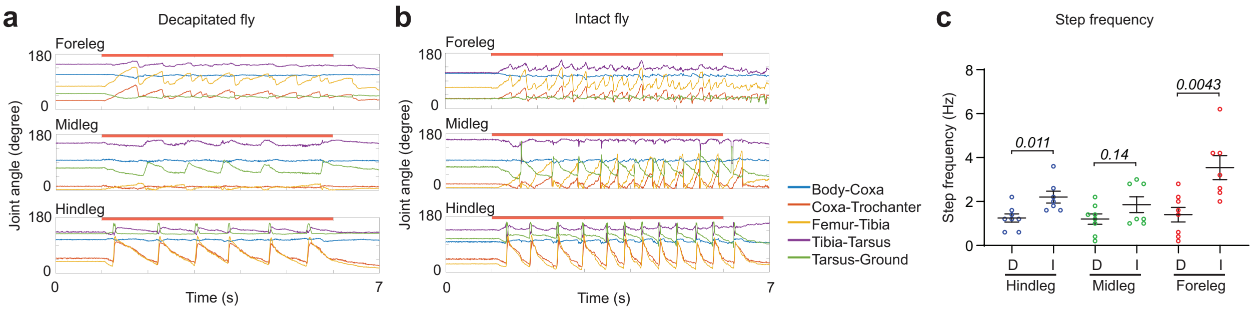

Supplementary Fig. 5. The backward walking program is largely intact in decapitated flies. **a** and **b**, Representative time series of joint angles in a decapitated (**a**) and intact (**b**) *MDN>CsChrimson* fly. Red bars indicate the 5-s red light stimulation. **c**, Backward step frequency for each leg in decapitated (D) and intact (I) flies $N = 7$ flies for each group. Error bars show mean ± s.e.m.. *P* values are shown in italics, two-tailed unpaired t-tests. Source data are provided as a Source Data file.

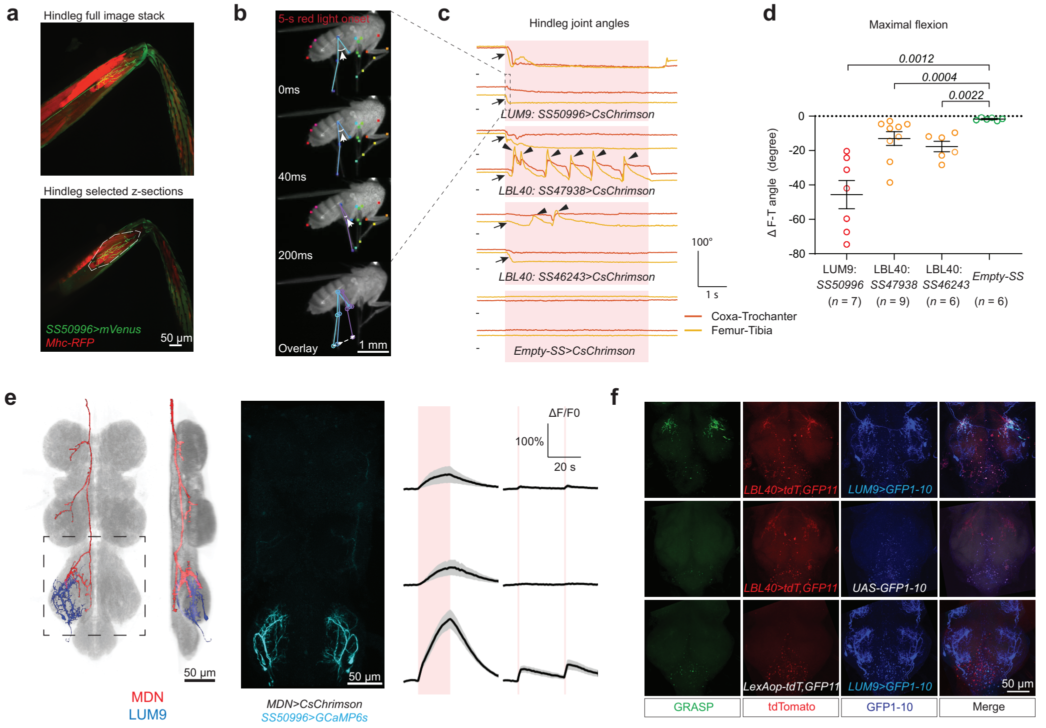

Supplementary Fig. 6. LBL40 triggers tibia flexion through LUM9. a, Top, maximum intensity projection of live fluorescence image of a hindleg, showing LUM9 innervation (mVenus, green) and all leg muscles (RFP, red). Bottom, selected z-slices highlighting innervation of the tibia reductor muscle (dashed line). **b**, Selected frames from a representative video of LUM9 activation in a suspended, decapitated fly, during a 5-s red light stimulus from t = 0 ms. Arrows indicate the femur-tibia angles. Dashed line with arrows in the overlay image indicates sequence of movement. Flies were suspended rather than placed on a ball because simultaneous activation of foreleg and hindleg tibia flexor muscles in a fly on a ball would generate opposing pulling forces, potentially resulting in little or no movement of either joint. **c**, Hindleg joint angle time series, showing two representative traces for each genotype. Red shade indicates a 5-s pulse of red light. A short black line marks the origin for each trace (t = 0 s, 0 degrees). Arrows indicate the initial femur-tibia flexion; arrowheads indicate extension events. The second set of traces is from the video shown in (**b**). **d**, Maximal femur-tibia joint angle change within 1 s after red light onset. Bars indicate mean ± s.e.m. *P* values are shown in italics, two-tailed Mann-Whitney tests. Source data are provided as a Source Data file. **e**, Left, registered segmented image of MDN (red), LUM9 (blue), and all synapses (gray, nc82). Scale bar: 50 μm. Middle, activated voxels (cyan) from calcium imaging of LUM9 upon MDN activation. Right, averaged responses for LUM9 to MDN activation (mean ± s.e.m., N = 7 flies), upon either a single 20-s stimulus or two 1-s light pulses (red shading). **f**, Live GFP fluorescence images showing reconstituted GFP (green) at likely synaptic connections between LBL40 (red, tdTomato live fluorescence, co-expressed with GFP11) and LUM9 (blue, anti-GFP staining against GFP1-10). The imaged areas correspond to the dashed box in **e**.

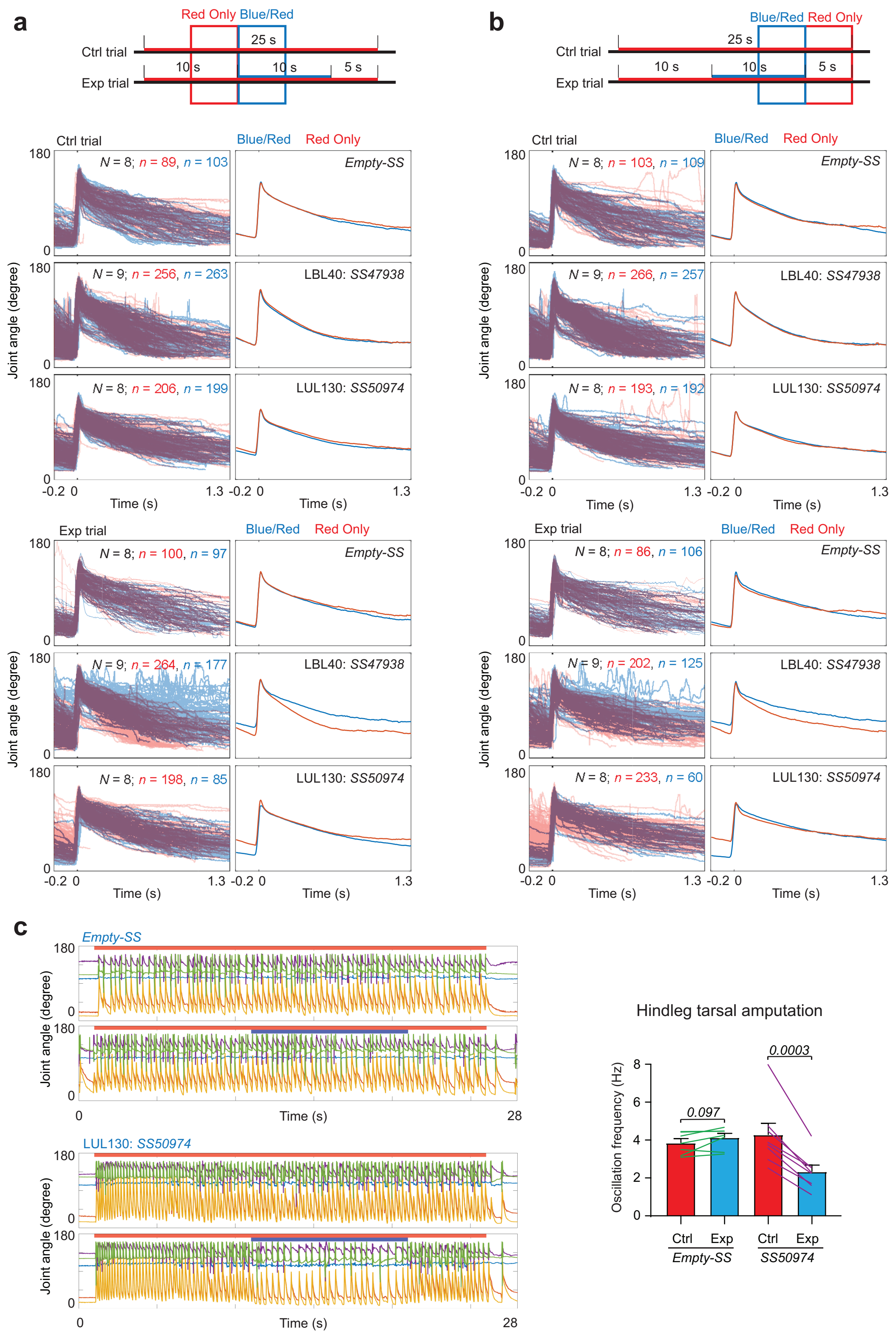
a
Red Only Blue/Red
25 s
Ctrl trial
10 s
10 s
5 s
Exp trial
b
Blue/Red Red Only
25 s
Ctrl trial
10 s
10 s
5 s
Exp trial
Ctrl trial
Blue/Red Red Only
N = 8; n = 89, n = 103
Empty-SS
N = 9; n = 256, n = 263
LBL40: SS47938
N = 8; n = 206, n = 199
LUL130: SS50974
N = 8; n = 103, n = 109
N = 9; n = 266, n = 257
N = 8; n = 193, n = 192
Joint angle (degree)
180
0
-0.2 0
1.3
Time (s)
Exp trial
Blue/Red Red Only
N = 8; n = 100, n = 97
N = 9; n = 264, n = 177
N = 8; n = 198, n = 85
N = 8; n = 86, n = 106
N = 9; n = 202, n = 125
N = 8; n = 233, n = 60
c
Empty-SS
LUL130: SS50974
Joint angle (degree)
Time (s)
0
28
Hindleg tarsal amputation
Oscillation frequency (Hz)
0.097
0.0003
Ctrl Exp
Empty-SS
Ctrl Exp
SS50974

Supplementary Fig. 7. Acute silencing of LBL40 or LUL130 leads to distinct defects in backward walking. a and **b**, Overlaid and average time series of femur-tibia joint angle for all the steps extracted from the indicated time windows. The same dataset as shown in Fig. 7 were analyzed using within-trial comparisons. **c**, Left, representative time series of joint angles in hindlegs amputated at tarsus. Red bar indicates red-light stimulus; blue bar indicates blue-light stimulus. Right, quantification of oscillation frequency of amputated hindlegs. $N = 7$ flies for *Empty-SS* and $N = 8$ for *SS50974*. Error bars show mean ± s.e.m. *P* values are shown in italics, two-tailed paired t-tests. Source data are provided as a Source Data file.

FULL GENOTYPE	SOURCE	SHORT GENOTYPE
w; VT044845-ZpGAL4DBD (attP40); VT050660-p65ADZp (attP2)	ref. 1	*MDN-1-GAL4*
w; VT049484-ZpGAL4DBD (attP40); VT050660-p65ADZp (attP2)	ref. 1	*MDN+MAN-1-GAL4*
w; VT044845-LexAp65 (attP40); +	ref. 2	*MDN-LexAp65*
w; VT049484-ZpLexADBD (JK22c); +	ref. 3	N/A
w; VT050660-p65ADZp (attP40); +	ref. 4	N/A
Vienna Tiles GAL4/LexA lines	ref. 4	See Supplementary Table 2
GMR GAL4/LexA lines	ref. 5	See Supplementary Table 2
Split GAL4 lines	refs. 4,6	See Supplementary Table 2
w; BPp65ADZpUw (attP40); BPZpGALDBDUw* (attP2)*	ref. 7	N/A
UAS-myrGFP,QUAS-mtdTomato(3xHA); trans-Tango; +	ref. 8	N/A
UAS-myrGFP, QUAS-mtdTomato(3xHA); trans-Tango, tubP-FRT-QS-FRT; +	ref. 8	N/A
w; ;MKRS, hsFLP86E/TM6B, Tb1	Bloomington Drosophila Stock Center (BDSC) 279	N/A
yw, UAS-myrGFP.QUAS-mtdTomato-3xHA (su(Hw)attP8), hs-FLP1; trans-Tango (attP40), FRT^{G13}*, tub-QS/CyO; +*	BDSC 77480	N/A
LexAop2-Syn21-opGCamp6s (su(HW)8), 10XUAS Syn21-Chrimson88-tdt3.1 (attP18); Sp/CyO; TM2/TM6	David Anderson, Barret Pfeiffer and Allan Wong	N/A
w; +; 13XLexAop2-CsChrimson-tdTomato (VK00005), 20XUAS Syn21-opGCaMP6s-p10-bp (su(Hw)attP1)	Vivek Jayaraman[9,10]	N/A
w; $VGlut^{LexA}$*; +*	ref. 11	N/A
R57C10-Flp2::PEST (attP18); brp::Snap; pJFRC201-10XUAS-FRT>STOP>FRT-myr::smGFP-HA (VK00005), pJFRC240-10XUAS-FRT>STOP>FRT-myr::smGFP-V5-THS-10XUAS-FRT>STOP>FRT-myr::smGFP-FLAG (su(Hw)attP1)	Gerry Rubin[12,13]	N/A
pJFRC300-20XUAS-FRT-dSTOP-FRTCsChrimson::mVenus (attP18), hs-FLP-PESTOpt (attP3); +; +	ref. 14	N/A
13XLexAop2-CsChrimson-tdTomato (attP18); +; +	Vivek Jayaraman[10]	N/A
20XUAS-CsChrimson-mVenus (attP18); +; +	Vivek Jayaraman[10]	N/A
w; UAS-TNT-E; +	ref. 15	N/A
w; +; UAS-GtACR2	ref. 16	N/A
w; CoinFLP-LexA::GAD-.GAL4 (attP40), LexAop-rCD2.RFP; UAS-CD4-spGFP1-10, LexAop-CD4-spGFP11/TM6C, Sb	BDSC 58755; ref. 17	N/A
w; ;13XLexAop2-IVS-myr::GFP (attP2)	ref. 18	N/A
w; pJFRC48-13XLexAop2-IVS-myrtdTomato (su(Hw)attP5); +	Gerry Rubin	N/A
yw, UAS-mCD8-GFP; Mhc-RFP; +	Richard Mann[19]	N/A

Supplementary Table 1 | Fly Stocks.

FIGURE	GENOTYPE	ABBREVIATED GENOTYPE
Fig. 1 and Supplementary Fig. 5	*20XUAS-CsChrimson-mVenus (attP18), w / w; V0T44845-ZpGAL4DBD(attP40) / +; VT050660-p65ADZp (attP2) / +*	N/A
Fig. 2a	*20XUAS-CsChrimson-mVenus (attP18), w / w, UAS-myrGFP, QUAS-mtdTomato(3xHA) (su(Hw)attP8); VT044845-ZpGAL4DBD (attP40) / trans-Tango ; VT050660-p65ADZp (attP2) / +*	trans-Tango
Fig. 2b, 2c and Supplementary Fig. 1	*20XUAS-CsChrimson-mVenus (attP18), w / yw, UAS-myrGFP, QUAS-mtdTomato(3xHA) (su(Hw)attP8), hsFLP1; VT044845-ZpGAL4DBD (attP40) / trans-Tango, tubP-FRT-QS-FRT; VT050660-p65ADZp (attP2) / +*	Stochastic trans-Tango
Fig. 2b, 2c and Supplementary Fig. 1	*20XUAS-CsChrimson-mVenus (attP18), w / w, UAS-myrGFP, QUAS-mtdTomato(3xHA) (su(Hw)attP8); VT044845-ZpGAL4DBD (attP40) / trans-Tango, tubP-FRT-QS-FRT; VT050660-p65ADZp (attP2) / MKRS, P{ry[+t7.2]=hsFLP}86E*	Stochastic trans-Tango
Fig. 3b	*LexAop2-Syn21-opGCamp6s (su(HW)8), 10XUAS Syn21-Chrimson88-tdt3.1 (attP18), w / w; VT044845-ZpGAL4DBD (attP40) / + ; VT050660-p65ADZp (attP2) / 57C10-LexA (attP2)*	*57C10>GCaMP6s*
Fig. 3b	*LexAop2-Syn21-opGCamp6s (su(HW)8), 10XUAS Syn21-Chrimson88-tdt3.1 (attP18), w / w; VT044845-ZpGAL4DBD (attP40) / VGlut*^{LexA}*; VT050660-p65ADZp (attP2) / +*	*VGlut>GCaMP6s*
Fig. 3d, Supplementary Fig. 2 and Fig. 5a	*w / w, VT049484-ZpLexADBD (JK22c) / +; VT050660-p65ADZp (attP2), 13XLexAop2-CsChrimson-tdTomato (VK00005), 20XUAS Syn21-opGCaMP6s-p10-bp (su(Hw)attP1) / VT029570-GAL4 (attP2)*	MF01
Fig. 3c,d and Supplementary Fig. 2	*LexAop2-Syn21-opGCamp6s (su(HW)8), 10XUAS Syn21-Chrimson88-tdt3.1 (attP18), w / w; VT044845-ZpGAL4DBD (attP40) / 28F07-LexA (attP40) ; VT050660-p65ADZp (attP2) / +*	MF02
Fig. 3d and Supplementary Fig. 2	*w / w, VT049484-ZpLexADBD (JK22c) / +; VT050660-p65ADZp (attP2), 13XLexAop2-CsChrimson-tdTomato (VK00005), 20XUAS Syn21-opGCaMP6s-p10-bp (su(Hw)attP1) / VT019902-GAL4 (attP2)*	MF03
Fig. 3d and Supplementary Fig. 2	*w / w, VT049484-ZpLexADBD (JK22c) / +; VT050660-p65ADZp (attP2), 13XLexAop2-CsChrimson-tdTomato (VK00005), 20XUAS Syn21-opGCaMP6s-p10-bp (su(Hw)attP1) / VT046303-GAL4 (attP2)*	MF04
Fig. 3d and Supplementary Fig. 2	*w / w, VT049484-ZpLexADBD (JK22c) / +; VT050660-p65ADZp (attP2), 13XLexAop2-CsChrimson-tdTomato (VK00005), 20XUAS Syn21-opGCaMP6s-p10-bp (su(Hw)attP1) / VT008473-GAL4 (attP2)*	MF05
Fig. 3d and Supplementary Fig. 2	*w / w, VT049484-ZpLexADBD (JK22c) / +; VT050660-p65ADZp (attP2), 13XLexAop2-CsChrimson-tdTomato (VK00005), 20XUAS Syn21-opGCaMP6s-p10-bp (su(Hw)attP1) / 92G01-GAL4 (attP2)*	MF06
Fig. 3d and Supplementary Fig. 2	*w / w, VT049484-ZpLexADBD (JK22c) / +; VT050660-p65ADZp (attP2), 13XLexAop2-CsChrimson-tdTomato (VK00005), 20XUAS Syn21-opGCaMP6s-p10-bp (su(Hw)attP1) / VT014974-GAL4 (attP2)*	MF07
Fig. 3d and Supplementary Fig. 2	*LexAop2-Syn21-opGCamp6s (su(HW)8), 10XUAS Syn21-Chrimson88-tdt3.1 (attP18), w / w; VT044845-ZpGAL4DBD (attP40) / VT043924-LexA (JK22c) ; VT050660-p65ADZp (attP2) / +*	MF08
Fig. 3d, Supplementary Fig. 2 and Fig. 6a	*w / w, VT049484-ZpLexADBD (JK22c) / +; VT050660-p65ADZp (attP2), 13XLexAop2-CsChrimson-tdTomato (VK00005), 20XUAS Syn21-opGCaMP6s-p10-bp (su(Hw)attP1) / VT062245-GAL4 (attP2)*	MF09
Fig. 3d and Supplementary Fig. 2	*w / w, VT049484-ZpLexADBD (JK22c) / +; VT050660-p65ADZp (attP2), 13XLexAop2-CsChrimson-tdTomato (VK00005), 20XUAS Syn21-opGCaMP6s-p10-bp (su(Hw)attP1) / VT002075-GAL4 (attP2)*	MF10
Fig. 3d and Supplementary Fig. 2	*LexAop2-Syn21-opGCamp6s (su(HW)8), 10XUAS Syn21-Chrimson88-tdt3.1 (attP18), w / w; VT044845-ZpGAL4DBD (attP40) / VT021842-LexA (attP40) ; VT050660-p65ADZp (attP2) / +*	MF11
Fig. 3d and Supplementary Fig. 2	*w / w, VT049484-ZpLexADBD (JK22c) / +; VT050660-p65ADZp (attP2), 13XLexAop2-CsChrimson-tdTomato (VK00005), 20XUAS Syn21-opGCaMP6s-p10-bp (su(Hw)attP1) / VT038219-GAL4 (attP2)*	MF12

Fig. 3d and Supplementary Fig. 2	*LexAop2-Syn21-opGCamp6s (su(HW)8), 10XUAS Syn21-Chrimson88-tdt3.1 (attP18), w / w; VT044845-ZpGAL4DBD (attP40) / 73B05-LexA (attP40) ; VT050660-p65ADZp (attP2) / +*	MF13
Fig. 3d and Supplementary Fig. 2	*w / w, VT049484-ZpLexADBD (JK22c) / +; VT050660-p65ADZp (attP2), 13XLexAop2-CsChrimson-tdTomato (VK00005), 20XUAS Syn21-opGCaMP6s-p10-bp (su(Hw)attP1) / VT002996-GAL4 (attP2)*	MF14
Fig. 3d and Supplementary Fig. 2	*w / w, VT049484-ZpLexADBD (JK22c) / +; VT050660-p65ADZp (attP2), 13XLexAop2-CsChrimson-tdTomato (VK00005), 20XUAS Syn21-opGCaMP6s-p10-bp (su(Hw)attP1) / VT019345-GAL4 (attP2)*	MF15
Fig. 3d and Supplementary Fig. 2	*w / w, VT049484-ZpLexADBD (JK22c) / +; VT050660-p65ADZp (attP2), 13XLexAop2-CsChrimson-tdTomato (VK00005), 20XUAS Syn21-opGCaMP6s-p10-bp (su(Hw)attP1) / 82F02-GAL4 (attP2)*	MF16
Fig. 3d and Supplementary Fig. 2	*LexAop2-Syn21-opGCamp6s (su(HW)8), 10XUAS Syn21-Chrimson88-tdt3.1 (attP18), w / w; VT044845-ZpGAL4DBD (attP40) / VT026669-LexA (attP40) ; VT050660-p65ADZp (attP2) / +*	MF17
Fig. 3d and Supplementary Fig. 2	*w / w, VT049484-ZpLexADBD (JK22c) / +; VT050660-p65ADZp (attP2), 13XLexAop2-CsChrimson-tdTomato (VK00005), 20XUAS Syn21-opGCaMP6s-p10-bp (su(Hw)attP1) / VT039419-GAL4 (attP2)*	MF18
Fig. 3d and Supplementary Fig. 2	*w / w, VT049484-ZpLexADBD (JK22c) / +; VT050660-p65ADZp (attP2), 13XLexAop2-CsChrimson-tdTomato (VK00005), 20XUAS Syn21-opGCaMP6s-p10-bp (su(Hw)attP1) / VT056582-GAL4 (attP2)*	MF19
Fig. 3d and Supplementary Fig. 2	*w / w, VT049484-ZpLexADBD (JK22c) / +; VT050660-p65ADZp (attP2), 13XLexAop2-CsChrimson-tdTomato (VK00005), 20XUAS Syn21-opGCaMP6s-p10-bp (su(Hw)attP1) / VT000353-GAL4 (attP2)*	MF20
Fig. 3d and Supplementary Fig. 2	*w / w, VT049484-ZpLexADBD (JK22c) / +; VT050660-p65ADZp (attP2), 13XLexAop2-CsChrimson-tdTomato (VK00005), 20XUAS Syn21-opGCaMP6s-p10-bp (su(Hw)attP1) / 29G03-GAL4 (attP2)*	MF21
Fig. 3d and Supplementary Fig. 2	*w / w, VT049484-ZpLexADBD (JK22c) / +; VT050660-p65ADZp (attP2), 13XLexAop2-CsChrimson-tdTomato (VK00005), 20XUAS Syn21-opGCaMP6s-p10-bp (su(Hw)attP1) / VT040713-GAL4 (attP2)*	MF22
Fig. 3d and Supplementary Fig. 2	*w / w, VT049484-ZpLexADBD (JK22c) / +; VT050660-p65ADZp (attP2), 13XLexAop2-CsChrimson-tdTomato (VK00005), 20XUAS Syn21-opGCaMP6s-p10-bp (su(Hw)attP1) / 19D12-GAL4 (attP2)*	MF23
Fig. 3d and Supplementary Fig. 2	*w / w, VT049484-ZpLexADBD (JK22c) / +; VT050660-p65ADZp (attP2), 13XLexAop2-CsChrimson-tdTomato (VK00005), 20XUAS Syn21-opGCaMP6s-p10-bp (su(Hw)attP1) / VT007722-GAL4 (attP2)*	MF24
Fig. 3e and Supplementary Fig. 2	*w / w, VT049484-ZpLexADBD (JK22c) / +; VT050660-p65ADZp (attP2), 13XLexAop2-CsChrimson-tdTomato (VK00005), 20XUAS Syn21-opGCaMP6s-p10-bp (su(Hw)attP1) / VT000254-GAL4 (attP2)*	MF25
Fig. 3e and Supplementary Fig. 2	*w / w, VT049484-ZpLexADBD (JK22c) / +; VT050660-p65ADZp (attP2), 13XLexAop2-CsChrimson-tdTomato (VK00005), 20XUAS Syn21-opGCaMP6s-p10-bp (su(Hw)attP1) / VT010555-GAL4 (attP2)*	MF26
Fig. 3e and Supplementary Fig. 2	*LexAop2-Syn21-opGCamp6s (su(HW)8), 10XUAS Syn21-Chrimson88-tdt3.1 (attP18), w / w; VT044845-ZpGAL4DBD (attP40) / +; VT050660-p65ADZp (attP2) / VT044492-LexA (attP2)*	MF27
Fig. 3e and Supplementary Fig. 2	*w / w, VT049484-ZpLexADBD (JK22c) / +; VT050660-p65ADZp (attP2), 13XLexAop2-CsChrimson-tdTomato (VK00005), 20XUAS Syn21-opGCaMP6s-p10-bp (su(Hw)attP1) / VT001497-GAL4 (attP2)*	MF28
Fig. 3e and Supplementary Fig. 2	*w / w, VT049484-ZpLexADBD (JK22c) / +; VT050660-p65ADZp (attP2), 13XLexAop2-CsChrimson-tdTomato (VK00005), 20XUAS Syn21-opGCaMP6s-p10-bp (su(Hw)attP1) / VT056808-GAL4 (attP2)*	MF29
Fig. 3e and Supplementary Fig. 2	*w / w, VT049484-ZpLexADBD (JK22c) / +; VT050660-p65ADZp (attP2), 13XLexAop2-CsChrimson-tdTomato (VK00005), 20XUAS Syn21-opGCaMP6s-p10-bp (su(Hw)attP1) / 22C10-GAL4 (attP2)*	MF30
Fig. 3e and Supplementary Fig. 2	*w / w, VT049484-ZpLexADBD (JK22c) / +; VT050660-p65ADZp (attP2), 13XLexAop2-CsChrimson-tdTomato (VK00005), 20XUAS Syn21-opGCaMP6s-p10-bp (su(Hw)attP1) / VT057467-GAL4 (attP2)*	MF31
Fig. 3e and Supplementary Fig. 2	*w / w, VT049484-ZpLexADBD (JK22c) / +; VT050660-p65ADZp (attP2), 13XLexAop2-CsChrimson-tdTomato (VK00005), 20XUAS Syn21-opGCaMP6s-p10-bp (su(Hw)attP1) / VT002223-GAL4 (attP2)*	MF32

Fig. 3f and Supplementary Fig. 2	*LexAop2-Syn21-opGCamp6s (su(HW)8), 10XUAS Syn21-Chrimson88-tdt3.1 (attP18), w / w; VT044845-ZpGAL4DBD (attP40) / 31H05-p65ADZp (attP40); VT050660-p65ADZp (attP2) / VT011123-ZpGAL4DBD (attP2)*	MF33
Fig. 3d and Supplementary Fig. 1	*R57C10-Flp2::PEST (attp18), w / w; brp::Snap / +; pJFRC201-10XUAS-FRT>STOP>FRT-myr::smGFP-HA (VK00005), pJFRC240-10XUAS-FRT>STOP>FRT-myr::smGFP-V5-THS-10XUAS-FRT>STOP>FRT-myr::smGFP-FLAG (su(Hw)attP1) / VT029570-GAL4 (attP2)*	MF01
Fig. 3c,d and Supplementary Fig. 1	*R57C10-Flp2::PEST (attp18), w / w; brp::Snap / +; pJFRC201-10XUAS-FRT>STOP>FRT-myr::smGFP-HA (VK00005), pJFRC240-10XUAS-FRT>STOP>FRT-myr::smGFP-V5-THS-10XUAS-FRT>STOP>FRT-myr::smGFP-FLAG (su(Hw)attP1) / 28F07-GAL4 (attP2)*	MF02
Fig. 3d and Supplementary Fig. 1	*R57C10-Flp2::PEST (attp18), w / w; brp::Snap / +; pJFRC201-10XUAS-FRT>STOP>FRT-myr::smGFP-HA (VK00005), pJFRC240-10XUAS-FRT>STOP>FRT-myr::smGFP-V5-THS-10XUAS-FRT>STOP>FRT-myr::smGFP-FLAG (su(Hw)attP1) / VT019902-GAL4 (attP2)*	MF03
Fig. 3d	*R57C10-Flp2::PEST (attp18), w / w; brp::Snap / +; pJFRC201-10XUAS-FRT>STOP>FRT-myr::smGFP-HA (VK00005), pJFRC240-10XUAS-FRT>STOP>FRT-myr::smGFP-V5-THS-10XUAS-FRT>STOP>FRT-myr::smGFP-FLAG (su(Hw)attP1) / VT046303-GAL4 (attP2)*	MF04
Fig. 3d	*R57C10-Flp2::PEST (attp18), w / w; brp::Snap / +; pJFRC201-10XUAS-FRT>STOP>FRT-myr::smGFP-HA (VK00005), pJFRC240-10XUAS-FRT>STOP>FRT-myr::smGFP-V5-THS-10XUAS-FRT>STOP>FRT-myr::smGFP-FLAG (su(Hw)attP1) / VT008473-GAL4 (attP2)*	MF05
Fig. 3d and Supplementary Fig. 1	*R57C10-Flp2::PEST (attp18), w / w; brp::Snap / +; pJFRC201-10XUAS-FRT>STOP>FRT-myr::smGFP-HA (VK00005), pJFRC240-10XUAS-FRT>STOP>FRT-myr::smGFP-V5-THS-10XUAS-FRT>STOP>FRT-myr::smGFP-FLAG (su(Hw)attP1) / 92G01-GAL4 (attP2)*	MF06
Fig. 3d	*R57C10-Flp2::PEST (attp18), w / w; brp::Snap / +; pJFRC201-10XUAS-FRT>STOP>FRT-myr::smGFP-HA (VK00005), pJFRC240-10XUAS-FRT>STOP>FRT-myr::smGFP-V5-THS-10XUAS-FRT>STOP>FRT-myr::smGFP-FLAG (su(Hw)attP1) / VT014974-GAL4 (attP2)*	MF07
Fig. 3d	*R57C10-Flp2::PEST (attp18), w / w; brp::Snap / +; pJFRC201-10XUAS-FRT>STOP>FRT-myr::smGFP-HA (VK00005), pJFRC240-10XUAS-FRT>STOP>FRT-myr::smGFP-V5-THS-10XUAS-FRT>STOP>FRT-myr::smGFP-FLAG (su(Hw)attP1) / VT043924-GAL4 (attP2)*	MF08
Fig. 3d and Supplementary Fig. 1	*R57C10-Flp2::PEST (attp18), w / w; brp::Snap / +; pJFRC201-10XUAS-FRT>STOP>FRT-myr::smGFP-HA (VK00005), pJFRC240-10XUAS-FRT>STOP>FRT-myr::smGFP-V5-THS-10XUAS-FRT>STOP>FRT-myr::smGFP-FLAG (su(Hw)attP1) / VT062245-GAL4 (attP2)*	MF09
Fig. 3d and Supplementary Fig. 1	*R57C10-Flp2::PEST (attp18), w / w; brp::Snap / +; pJFRC201-10XUAS-FRT>STOP>FRT-myr::smGFP-HA (VK00005), pJFRC240-10XUAS-FRT>STOP>FRT-myr::smGFP-V5-THS-10XUAS-FRT>STOP>FRT-myr::smGFP-FLAG (su(Hw)attP1) / VT002075-GAL4 (attP2)*	MF10
Fig. 3d	*R57C10-Flp2::PEST (attp18), w / w; brp::Snap / +; pJFRC201-10XUAS-FRT>STOP>FRT-myr::smGFP-HA (VK00005), pJFRC240-10XUAS-FRT>STOP>FRT-myr::smGFP-V5-THS-10XUAS-FRT>STOP>FRT-myr::smGFP-FLAG (su(Hw)attP1) / VT021842-GAL4 (attP2)*	MF11
Fig. 3d and Supplementary Fig. 1	*R57C10-Flp2::PEST (attp18), w / w; brp::Snap / +; pJFRC201-10XUAS-FRT>STOP>FRT-myr::smGFP-HA (VK00005), pJFRC240-10XUAS-FRT>STOP>FRT-myr::smGFP-V5-THS-10XUAS-FRT>STOP>FRT-myr::smGFP-FLAG (su(Hw)attP1) / VT038219-GAL4 (attP2)*	MF12
Fig. 3d and Supplementary Fig. 1	*R57C10-Flp2::PEST (attp18), w / w; 73B05-p65ADZp (attp40); 42H01-ZpGAL4DBD (attp2), pJFRC201-10XUAS-FRT>STOP>FRT-myr::smGFP-HA (VK00005), pJFRC240-10XUAS-FRT>STOP>FRT-myr::smGFP-V5-THS-10XUAS-FRT>STOP>FRT-myr::smGFP-FLAG (su(Hw)attP1)*	MF13
Fig. 3d	*R57C10-Flp2::PEST (attp18), w / w; brp::Snap / +; pJFRC201-10XUAS-FRT>STOP>FRT-myr::smGFP-HA (VK00005), pJFRC240-10XUAS-FRT>STOP>FRT-myr::smGFP-V5-THS-10XUAS-FRT>STOP>FRT-myr::smGFP-FLAG (su(Hw)attP1) / VT002996-GAL4 (attP2)*	MF14
Fig. 3d	*R57C10-Flp2::PEST (attp18), w / w; brp::Snap / +; pJFRC201-10XUAS-FRT>STOP>FRT-myr::smGFP-HA (VK00005), pJFRC240-10XUAS-*	MF15

	FRT>STOP>FRT-myr::smGFP-V5-THS-10XUAS-FRT>STOP>FRT-myr::smGFP-FLAG (su(Hw)attP1) / VT019345-GAL4 (attP2)	
Fig. 3d and Supplementary Fig. 1	*R57C10-Flp2::PEST (attp18), w / w; brp::Snap / +; pJFRC201-10XUAS-FRT>STOP>FRT-myr::smGFP-HA (VK00005), pJFRC240-10XUAS-FRT>STOP>FRT-myr::smGFP-V5-THS-10XUAS-FRT>STOP>FRT-myr::smGFP-FLAG (su(Hw)attP1) / 82F02-GAL4 (attP2)*	MF16
Fig. 3d and Supplementary Fig. 1	*R57C10-Flp2::PEST (attp18), w / w; brp::Snap / +; pJFRC201-10XUAS-FRT>STOP>FRT-myr::smGFP-HA (VK00005), pJFRC240-10XUAS-FRT>STOP>FRT-myr::smGFP-V5-THS-10XUAS-FRT>STOP>FRT-myr::smGFP-FLAG (su(Hw)attP1) / VT026669-GAL4 (attP2)*	MF17
Fig. 3d and Supplementary Fig. 1	*R57C10-Flp2::PEST (attp18), w / w; brp::Snap / +; pJFRC201-10XUAS-FRT>STOP>FRT-myr::smGFP-HA (VK00005), pJFRC240-10XUAS-FRT>STOP>FRT-myr::smGFP-V5-THS-10XUAS-FRT>STOP>FRT-myr::smGFP-FLAG (su(Hw)attP1) / VT039419-GAL4 (attP2)*	MF18
Fig. 3d and Supplementary Fig. 1	*R57C10-Flp2::PEST (attp18), w / w; brp::Snap / +; pJFRC201-10XUAS-FRT>STOP>FRT-myr::smGFP-HA (VK00005), pJFRC240-10XUAS-FRT>STOP>FRT-myr::smGFP-V5-THS-10XUAS-FRT>STOP>FRT-myr::smGFP-FLAG (su(Hw)attP1) / VT056582-GAL4 (attP2)*	MF19
Fig. 3d and Supplementary Fig. 1	*R57C10-Flp2::PEST (attp18), w / w; brp::Snap / +; pJFRC201-10XUAS-FRT>STOP>FRT-myr::smGFP-HA (VK00005), pJFRC240-10XUAS-FRT>STOP>FRT-myr::smGFP-V5-THS-10XUAS-FRT>STOP>FRT-myr::smGFP-FLAG (su(Hw)attP1) / VT000353-GAL4 (attP2)*	MF20
Fig. 3d	*R57C10-Flp2::PEST (attp18), w / w; brp::Snap / +; pJFRC201-10XUAS-FRT>STOP>FRT-myr::smGFP-HA (VK00005), pJFRC240-10XUAS-FRT>STOP>FRT-myr::smGFP-V5-THS-10XUAS-FRT>STOP>FRT-myr::smGFP-FLAG (su(Hw)attP1) / 29G03-GAL4 (attP2)*	MF21
Fig. 3d and Supplementary Fig. 1	*R57C10-Flp2::PEST (attp18), w / w; brp::Snap / +; pJFRC201-10XUAS-FRT>STOP>FRT-myr::smGFP-HA (VK00005), pJFRC240-10XUAS-FRT>STOP>FRT-myr::smGFP-V5-THS-10XUAS-FRT>STOP>FRT-myr::smGFP-FLAG (su(Hw)attP1) / VT040713-GAL4 (attP2)*	MF22
Fig. 3d and Supplementary Fig. 1	*R57C10-Flp2::PEST (attp18), w / w; brp::Snap / +; pJFRC201-10XUAS-FRT>STOP>FRT-myr::smGFP-HA (VK00005), pJFRC240-10XUAS-FRT>STOP>FRT-myr::smGFP-V5-THS-10XUAS-FRT>STOP>FRT-myr::smGFP-FLAG (su(Hw)attP1) / 19D12-GAL4 (attP2)*	MF23
Fig. 3d and Supplementary Fig. 1	*R57C10-Flp2::PEST (attp18), w / w; brp::Snap / +; pJFRC201-10XUAS-FRT>STOP>FRT-myr::smGFP-HA (VK00005), pJFRC240-10XUAS-FRT>STOP>FRT-myr::smGFP-V5-THS-10XUAS-FRT>STOP>FRT-myr::smGFP-FLAG (su(Hw)attP1) / VT007722-GAL4 (attP2)*	MF24
Fig. 3e	*R57C10-Flp2::PEST (attp18), w / w; brp::Snap / +; pJFRC201-10XUAS-FRT>STOP>FRT-myr::smGFP-HA (VK00005), pJFRC240-10XUAS-FRT>STOP>FRT-myr::smGFP-V5-THS-10XUAS-FRT>STOP>FRT-myr::smGFP-FLAG (su(Hw)attP1) / VT000254-GAL4 (attP2)*	MF25
Fig. 3e and Supplementary Fig. 1	*R57C10-Flp2::PEST (attp18), w / w; brp::Snap / +; pJFRC201-10XUAS-FRT>STOP>FRT-myr::smGFP-HA (VK00005), pJFRC240-10XUAS-FRT>STOP>FRT-myr::smGFP-V5-THS-10XUAS-FRT>STOP>FRT-myr::smGFP-FLAG (su(Hw)attP1) / VT010555-GAL4 (attP2)*	MF26
Fig. 3e	*R57C10-Flp2::PEST (attp18), w / w; brp::Snap / +; pJFRC201-10XUAS-FRT>STOP>FRT-myr::smGFP-HA (VK00005), pJFRC240-10XUAS-FRT>STOP>FRT-myr::smGFP-V5-THS-10XUAS-FRT>STOP>FRT-myr::smGFP-FLAG (su(Hw)attP1) / VT044492-GAL4 (attP2)*	MF27
Fig. 3e and Supplementary Fig. 1	*R57C10-Flp2::PEST (attp18), w / w; brp::Snap / +; pJFRC201-10XUAS-FRT>STOP>FRT-myr::smGFP-HA (VK00005), pJFRC240-10XUAS-FRT>STOP>FRT-myr::smGFP-V5-THS-10XUAS-FRT>STOP>FRT-myr::smGFP-FLAG (su(Hw)attP1) / VT001497-GAL4 (attP2)*	MF28
Fig. 3e	*R57C10-Flp2::PEST (attp18), w / w; brp::Snap / +; pJFRC201-10XUAS-FRT>STOP>FRT-myr::smGFP-HA (VK00005), pJFRC240-10XUAS-FRT>STOP>FRT-myr::smGFP-V5-THS-10XUAS-FRT>STOP>FRT-myr::smGFP-FLAG (su(Hw)attP1) / VT056808-GAL4 (attP2)*	MF29
Fig. 3e and Supplementary Fig. 1	*R57C10-Flp2::PEST (attp18), w / w; brp::Snap / +; pJFRC201-10XUAS-FRT>STOP>FRT-myr::smGFP-HA (VK00005), pJFRC240-10XUAS-*	MF30

	FRT>STOP>FRT-myr::smGFP-V5-THS-10XUAS-FRT>STOP>FRT-myr::smGFP-FLAG (su(Hw)attP1) / 22c10-GAL4 (attP2)	
Fig. 3e	R57C10-Flp2::PEST (attp18), w / w; brp::Snap / +; pJFRC201-10XUAS-FRT>STOP>FRT-myr::smGFP-HA (VK00005), pJFRC240-10XUAS-FRT>STOP>FRT-myr::smGFP-V5-THS-10XUAS-FRT>STOP>FRT-myr::smGFP-FLAG (su(Hw)attP1) / VT057467-GAL4 (attP2)	MF31
Fig. 3e and Supplementary Fig. 1	R57C10-Flp2::PEST (attp18), w / w; brp::Snap / +; pJFRC201-10XUAS-FRT>STOP>FRT-myr::smGFP-HA (VK00005), pJFRC240-10XUAS-FRT>STOP>FRT-myr::smGFP-V5-THS-10XUAS-FRT>STOP>FRT-myr::smGFP-FLAG (su(Hw)attP1) / VT002223-GAL4 (attP2)	MF32
Fig. 3f	R57C10-Flp2::PEST (attp18), w / w; 31H05-p65ADZp (attp40) / +; VT011123-ZpGAL4DBD (attp2), pJFRC201-10XUAS-FRT>STOP>FRT-myr::smGFP-HA (VK00005), pJFRC240-10XUAS-FRT>STOP>FRT-myr::smGFP-V5-THS-10XUAS-FRT>STOP>FRT-myr::smGFP-FLAG (su(Hw)attP1)	MF33
Supplementary Fig. 2	LexAop2-Syn21-opGCamp6s (su(HW)8), 10XUAS Syn21-Chrimson88-tdt3.1 (attP18), w / w; VT044845-ZpGAL4DBD (attP40) / 70B03-LexA (attP40) ; VT050660-p65ADZp (attP2) / +	MF14 (70B03)
Supplementary Fig. 2	w / w, VT049484-ZpLexADBD (JK22c) / +; VT050660-p65ADZp (attP2), 13XLexAop2-CsChrimson-tdTomato (VK00005), 20XUAS Syn21-opGCaMP6s-p10-bp (su(Hw)attP1) / VT048580-GAL4 (attP2)	MF30 (VT048580)
Supplementary Fig. 2	LexAop2-Syn21-opGCamp6s (su(HW)8), 10XUAS Syn21-Chrimson88-tdt3.1 (attP18), w / w; VT044845-ZpGAL4DBD (attP40) / VT048146-LexA (attP40) ; VT050660-p65ADZp (attP2) / +	MF15 (VT048146)
Supplementary Fig. 2	LexAop2-Syn21-opGCamp6s (su(HW)8), 10XUAS Syn21-Chrimson88-tdt3.1 (attP18), w / w; VT044845-ZpGAL4DBD (attP40) / VT044964-LexA (attP40) ; VT050660-p65ADZp (attP2) / +	MF08 (VT044964)
Supplementary Fig. 2	w / w, VT049484-ZpLexADBD (JK22c) / +; VT050660-p65ADZp (attP2), 13XLexAop2-CsChrimson-tdTomato (VK00005), 20XUAS Syn21-opGCaMP6s-p10-bp (su(Hw)attP1) / VT030541-GAL4 (attP2)	MF16 (VT030541)
Supplementary Fig. 2	LexAop2-Syn21-opGCamp6s (su(HW)8), 10XUAS Syn21-Chrimson88-tdt3.1 (attP18), w / w; VT044845-ZpGAL4DBD (attP40) / VT026669-LexA (attP40) ; VT050660-p65ADZp (attP2) / +	MF04 (VT026669)
Fig. 4b and Supplementary Fig. 3b	w / w, UAS-TNTe, VT049484-ZpLexADBD (JK22c) / pBPp65ADZpUw* (attP40); VT050660-p65ADZp (attP2), 13XLexAop2-CsChrimson-tdTomato (VK00005) / pBPZpGAL4DBDUw* (attP2)	Empty-SS
Fig. 4b and Supplementary Fig. 3b	w / w, UAS-TNTe, VT049484-ZpLexADBD (JK22c) / VT044845-p65ADZp (attP40); VT050660-p65ADZp (attP2), 13XLexAop2-CsChrimson-tdTomato (VK00005) / VT050660-p65ADZp (attP2)	MDN-1
Fig. 4b and Supplementary Fig. 3b	w / w, UAS-TNTe, VT049484-ZpLexADBD (JK22c) / VT050660-p65ADZp (attP40); VT050660-p65ADZp (attP2), 13XLexAop2-CsChrimson-tdTomato (VK00005) / VT049484-ZpGAL4DBD (attP2)	MDN+MAN-1
Fig. 4b and Supplementary Fig. 3b	w / w, UAS-TNTe, VT049484-ZpLexADBD (JK22c) / 73B05-p65ADZp (attP40); VT050660-p65ADZp (attP2), 13XLexAop2-CsChrimson-tdTomato (VK00005) / 42H01-ZpGAL4DBD (attP2)	SS27416
Fig. 4b and Supplementary Fig. 3b	w / w, UAS-TNTe, VT049484-ZpLexADBD (JK22c) / 72E10-p65ADZp (attP40); VT050660-p65ADZp (attP2), 13XLexAop2-CsChrimson-tdTomato (VK00005) / VT049904-ZpGAL4DBD (attP2)	SS32496
Fig. 4b and Supplementary Fig. 3b	w / w, UAS-TNTe, VT049484-ZpLexADBD (JK22c) / VT043924-p65ADZp (attP40); VT050660-p65ADZp (attP2), 13XLexAop2-CsChrimson-tdTomato (VK00005) / VT044964-ZpGAL4DBD (attP2)	SS33343
Fig. 4b and Supplementary Fig. 3b	w / w, UAS-TNTe, VT049484-ZpLexADBD (JK22c) / VT033629-p65ADZp (attP40); VT050660-p65ADZp (attP2), 13XLexAop2-CsChrimson-tdTomato (VK00005) / VT021799-ZpGAL4DBD (attP2)	SS33510
Fig. 4b and Supplementary Fig. 3b	w / w, UAS-TNTe, VT049484-ZpLexADBD (JK22c) / VT044964-p65ADZp (attP40); VT050660-p65ADZp (attP2), 13XLexAop2-CsChrimson-tdTomato (VK00005) / VT023795-ZpGAL4DBD (attP2)	SS33542
Fig. 4b and Supplementary Fig. 3b	w / w, UAS-TNTe, VT049484-ZpLexADBD (JK22c) / 77B09-p65ADZp (attP40); VT050660-p65ADZp (attP2), 13XLexAop2-CsChrimson-tdTomato (VK00005) / 72c08-ZpGAL4DBD (attP2)	SS35560

Fig. 4b and Supplementary Fig. 3b	*w / w, UAS-TNTe, VT049484-ZpLexADBD (JK22c) / 92A10-p65ADZp (attP40); VT050660-p65ADZp (attP2), 13XLexAop2-CsChrimson-tdTomato (VK00005) / VT019902-ZpGAL4DBD (attP2)*	*SS36194*
Fig. 4b and Supplementary Fig. 3b	*w / w, UAS-TNTe, VT049484-ZpLexADBD (JK22c) / VT001608-p65ADZp (attP40); VT050660-p65ADZp (attP2), 13XLexAop2-CsChrimson-tdTomato (VK00005) / VT025916-ZpGAL4DBD (attP2)*	*SS38205*
Fig. 4b and Supplementary Fig. 3b	*w / w, UAS-TNTe, VT049484-ZpLexADBD (JK22c) / VT063303-p65ADZp (attP40); VT050660-p65ADZp (attP2), 13XLexAop2-CsChrimson-tdTomato (VK00005) / VT063741-ZpGAL4DBD (attP2)*	*SS39036*
Fig. 4b and Supplementary Fig. 3b	*w / w, UAS-TNTe, VT049484-ZpLexADBD (JK22c) / 78A01-p65ADZp (attP40); VT050660-p65ADZp (attP2), 13XLexAop2-CsChrimson-tdTomato (VK00005) / VT009084-ZpGAL4DBD (attP2)*	*SS43697*
Fig. 4b and Supplementary Fig. 3b	*w / w, UAS-TNTe, VT049484-ZpLexADBD (JK22c) / VT064563-p65ADZp (attP40); VT050660-p65ADZp (attP2), 13XLexAop2-CsChrimson-tdTomato (VK00005) / VT000254-ZpGAL4DBD (attP2)*	*SS44225*
Fig. 4b and Supplementary Fig. 3b	*w / w, UAS-TNTe, VT049484-ZpLexADBD (JK22c) / VT065322-p65ADZp (attP40); VT050660-p65ADZp (attP2), 13XLexAop2-CsChrimson-tdTomato (VK00005) / VT019902-ZpGAL4DBD (attP2)*	*SS44254*
Fig. 4b and Supplementary Fig. 3b	*w / w, UAS-TNTe, VT049484-ZpLexADBD (JK22c) / VT065322-p65ADZp (attP40); VT050660-p65ADZp (attP2), 13XLexAop2-CsChrimson-tdTomato (VK00005) / 92A10-ZpGAL4DBD (attP2)*	*SS44255*
Fig. 4b and Supplementary Fig. 3b	*w / w, UAS-TNTe, VT049484-ZpLexADBD (JK22c) / VT005404-p65ADZp (attP40); VT050660-p65ADZp (attP2), 13XLexAop2-CsChrimson-tdTomato (VK00005) / VT000254-ZpGAL4DBD (attP2)*	*SS46225*
Fig. 4b and Supplementary Fig. 3b	*w / w, UAS-TNTe, VT049484-ZpLexADBD (JK22c) / VT033616-p65ADZp (attP40); VT050660-p65ADZp (attP2), 13XLexAop2-CsChrimson-tdTomato (VK00005) / VT008473-ZpGAL4DBD (attP2)*	*SS46229*
Fig. 4b and Supplementary Fig. 3b	*w / w, UAS-TNTe, VT049484-ZpLexADBD (JK22c) / VT029814-p65ADZp (attP40); VT050660-p65ADZp (attP2), 13XLexAop2-CsChrimson-tdTomato (VK00005) / VT028464-ZpGAL4DBD (attP2)*	*SS46233*
Fig. 4b and Supplementary Fig. 3b	*w / w, UAS-TNTe, VT049484-ZpLexADBD (JK22c) / VT006480-p65ADZp (attP40); VT050660-p65ADZp (attP2), 13XLexAop2-CsChrimson-tdTomato (VK00005) / 24A02-ZpGAL4DBD (attP2)*	*SS46243*
Fig. 4b and Supplementary Fig. 3b	*w / w, UAS-TNTe, VT049484-ZpLexADBD (JK22c) / 24A02-p65ADZp (attP40); VT050660-p65ADZp (attP2), 13XLexAop2-CsChrimson-tdTomato (VK00005) / VT006480-ZpGAL4DBD (attP2)*	*SS46253*
Fig. 4b and Supplementary Fig. 3b	*w / w, UAS-TNTe, VT049484-ZpLexADBD (JK22c) / 31H09-p65ADZp (attP40); VT050660-p65ADZp (attP2), 13XLexAop2-CsChrimson-tdTomato (VK00005) / VT020829-ZpGAL4DBD (attP2)*	*SS46258*
Fig. 4b and Supplementary Fig. 3b	*w / w, UAS-TNTe, VT049484-ZpLexADBD (JK22c) / VT043146-p65ADZp (attP40); VT050660-p65ADZp (attP2), 13XLexAop2-CsChrimson-tdTomato (VK00005) / VT000254-ZpGAL4DBD (attP2)*	*SS46300*
Fig. 4b and Supplementary Fig. 3b	*w / w, UAS-TNTe, VT049484-ZpLexADBD (JK22c) / VT026777-p65ADZp (attP40); VT050660-p65ADZp (attP2), 13XLexAop2-CsChrimson-tdTomato (VK00005) / VT049125-ZpGAL4DBD (attP2)*	*SS46669*
Fig. 4b and Supplementary Fig. 3b	*w / w, UAS-TNTe, VT049484-ZpLexADBD (JK22c) / VT037566-p65ADZp (attP40); VT050660-p65ADZp (attP2), 13XLexAop2-CsChrimson-tdTomato (VK00005) / VT008808-ZpGAL4DBD (attP2)*	*SS46730*
Fig. 4b and Supplementary Fig. 3b	*w / w, UAS-TNTe, VT049484-ZpLexADBD (JK22c) / VT058873-p65ADZp (attP40); VT050660-p65ADZp (attP2), 13XLexAop2-CsChrimson-tdTomato (VK00005) / VT058566-ZpGAL4DBD (attP2)*	*SS47847*
Fig. 4b and Supplementary Fig. 3b	*w / w, UAS-TNTe, VT049484-ZpLexADBD (JK22c) / 15D08-p65ADZp (attP40); VT050660-p65ADZp (attP2), 13XLexAop2-CsChrimson-tdTomato (VK00005) / 72E10-ZpGAL4DBD (attP2)*	*SS47863*
Fig. 4b and Supplementary Fig. 3b	*w / w, UAS-TNTe, VT049484-ZpLexADBD (JK22c) / VT021418-p65ADZp (attP40); VT050660-p65ADZp (attP2), 13XLexAop2-CsChrimson-tdTomato (VK00005) / 24A02-ZpGAL4DBD (attP2)*	*SS47921*
Fig. 4b and Supplementary Fig. 3b	*w / w, UAS-TNTe, VT049484-ZpLexADBD (JK22c) / 24A02-p65ADZp (attP40); VT050660-p65ADZp (attP2), 13XLexAop2-CsChrimson-tdTomato (VK00005) / VT021418-ZpGAL4DBD (attP2)*	*SS47938*

Fig. 4b and Supplementary Fig. 3b	*w / w, UAS-TNTe, VT049484-ZpLexADBD (JK22c) / VT008808-p65ADZp (attP40); VT050660-p65ADZp (attP2), 13XLexAop2-CsChrimson-tdTomato (VK00005) / VT048646-ZpGAL4DBD (attP2)*	*SS48376*
Fig. 4b and Supplementary Fig. 3b	*w / w, UAS-TNTe, VT049484-ZpLexADBD (JK22c) / 75B10-p65ADZp (attP40); VT050660-p65ADZp (attP2), 13XLexAop2-CsChrimson-tdTomato (VK00005) / 94E11-ZpGAL4DBD (attP2)*	*SS48718*
Fig. 4b and Supplementary Fig. 3b	*w / w, UAS-TNTe, VT049484-ZpLexADBD (JK22c) / VT062245-p65ADZp (attP40); VT050660-p65ADZp (attP2), 13XLexAop2-CsChrimson-tdTomato (VK00005) / VT050105-ZpGAL4DBD (attP2)*	*SS50974*
Fig. 4b and Supplementary Fig. 3b	*w / w, UAS-TNTe, VT049484-ZpLexADBD (JK22c) / VT062245-p65ADZp (attP40); VT050660-p65ADZp (attP2), 13XLexAop2-CsChrimson-tdTomato (VK00005) / VT021374-ZpGAL4DBD (attP2)*	*SS50975*
Fig. 4b and Supplementary Fig. 3b	*w / w, UAS-TNTe, VT049484-ZpLexADBD (JK22c) / 24A02-p65ADZp (attP40); VT050660-p65ADZp (attP2), 13XLexAop2-CsChrimson-tdTomato (VK00005) / VT000360-ZpGAL4DBD (attP2)*	*SS50995*
Fig. 4b and Supplementary Fig. 3b	*w / w, UAS-TNTe, VT049484-ZpLexADBD (JK22c) / 31H05-p65ADZp (attP40); VT050660-p65ADZp (attP2), 13XLexAop2-CsChrimson-tdTomato (VK00005) / VT011123-ZpGAL4DBD (attP2)*	*SS50996*
Fig. 4b and Supplementary Fig. 3b	*w / w, UAS-TNTe, VT049484-ZpLexADBD (JK22c) / VT024602-p65ADZp (attP40); VT050660-p65ADZp (attP2), 13XLexAop2-CsChrimson-tdTomato (VK00005) / VT021374-ZpGAL4DBD (attP2)*	*SS51817*
Fig. 4b and Supplementary Fig. 3b	*w / w, UAS-TNTe, VT049484-ZpLexADBD (JK22c) / VT021418-p65ADZp (attP40); VT050660-p65ADZp (attP2), 13XLexAop2-CsChrimson-tdTomato (VK00005) / VT012717-ZpGAL4DBD (attP2)*	*SS51843*
Fig. 4b and Supplementary Fig. 3b	*w / w, UAS-TNTe, VT049484-ZpLexADBD (JK22c) / 23G01-p65ADZp (attP40); VT050660-p65ADZp (attP2), 13XLexAop2-CsChrimson-tdTomato (VK00005) / VT037810-ZpGAL4DBD (attP2)*	*SS51877*
Fig. 4b and Supplementary Fig. 3b	*w / w, UAS-TNTe, VT049484-ZpLexADBD (JK22c) / 28F07-p65ADZp (attP40); VT050660-p65ADZp (attP2), 13XLexAop2-CsChrimson-tdTomato (VK00005) / VT008808-ZpGAL4DBD (attP2)*	*SS51891*
Fig. 4b and Supplementary Fig. 3b	*w / w, UAS-TNTe, VT049484-ZpLexADBD (JK22c) / 47A08-p65ADZp (attP40); VT050660-p65ADZp (attP2), 13XLexAop2-CsChrimson-tdTomato (VK00005) / VT029814-ZpGAL4DBD (attP2)*	*SS51895*
Fig. 4b and Supplementary Fig. 3b	*w / w, UAS-TNTe, VT049484-ZpLexADBD (JK22c) / 70E04-p65ADZp (attP40); VT050660-p65ADZp (attP2), 13XLexAop2-CsChrimson-tdTomato (VK00005) / VT021418-ZpGAL4DBD (attP2)*	*SS51907*
Fig. 4b and Supplementary Fig. 3b	*w / w, UAS-TNTe, VT049484-ZpLexADBD (JK22c) / 24E12-p65ADZp (attP40); VT050660-p65ADZp (attP2), 13XLexAop2-CsChrimson-tdTomato (VK00005) / VT019345-ZpGAL4DBD (attP2)*	*SS53029*
Fig. 4b and Supplementary Fig. 3b	*w / w, UAS-TNTe, VT049484-ZpLexADBD (JK22c) / 24E12-p65ADZp (attP40); VT050660-p65ADZp (attP2), 13XLexAop2-CsChrimson-tdTomato (VK00005) / VT048146-ZpGAL4DBD (attP2)*	*SS53030*
Fig. 4b and Supplementary Fig. 3b	*w / w, UAS-TNTe, VT049484-ZpLexADBD (JK22c) / 81A04-p65ADZp (attP40); VT050660-p65ADZp (attP2), 13XLexAop2-CsChrimson-tdTomato (VK00005) / 81A06-ZpGAL4DBD (attP2)*	*SS53050*
Fig. 4b and Supplementary Fig. 3b	*w / w, UAS-TNTe, VT049484-ZpLexADBD (JK22c) / VT002081-p65ADZp (attP40); VT050660-p65ADZp (attP2), 13XLexAop2-CsChrimson-tdTomato (VK00005) / VT019345-ZpGAL4DBD (attP2)*	*SS53903*
Fig. 4b and Supplementary Fig. 3b	*w / w, UAS-TNTe, VT049484-ZpLexADBD (JK22c) / VT029814-p65ADZp (attP40); VT050660-p65ADZp (attP2), 13XLexAop2-CsChrimson-tdTomato (VK00005) / VT049340-ZpGAL4DBD (attP2)*	*SS57939*
Fig. 4b and Supplementary Fig. 3b	*w / w, UAS-TNTe, VT049484-ZpLexADBD (JK22c) / VT049362-p65ADZp (attP40); VT050660-p65ADZp (attP2), 13XLexAop2-CsChrimson-tdTomato (VK00005) / VT021374-ZpGAL4DBD (attP2)*	*SS57941*
Fig. 4b and Supplementary Fig. 3b	*w / w, UAS-TNTe, VT049484-ZpLexADBD (JK22c) / VT029814-p65ADZp (attP40); VT050660-p65ADZp (attP2), 13XLexAop2-CsChrimson-tdTomato (VK00005) / VT025717-ZpGAL4DBD (attP2)*	*SS57954*
Fig. 4b and Supplementary Fig. 3b	*w / w, UAS-TNTe, VT049484-ZpLexADBD (JK22c) / VT019307-p65ADZp (attP40); VT050660-p65ADZp (attP2), 13XLexAop2-CsChrimson-tdTomato (VK00005) / VT025717-ZpGAL4DBD (attP2)*	*SS57955*

Fig. 4b and Supplementary Fig. 3b	*w / w, UAS-TNTe, VT049484-ZpLexADBD (JK22c) / VT062633-p65ADZp (attP40); VT050660-p65ADZp (attP2), 13XLexAop2-CsChrimson-tdTomato (VK00005) / VT008808-ZpGAL4DBD (attP2)*	*SS57977*
Fig. 4b and Supplementary Fig. 3b	*w / w, UAS-TNTe, VT049484-ZpLexADBD (JK22c) / VT062633-p65ADZp (attP40); VT050660-p65ADZp (attP2), 13XLexAop2-CsChrimson-tdTomato (VK00005) / 28F07-ZpGAL4DBD (attP2)*	*SS58000*
Fig. 4b and Supplementary Fig. 3b	*w / w, UAS-TNTe, VT049484-ZpLexADBD (JK22c) / VT045608-p65ADZp (attP40); VT050660-p65ADZp (attP2), 13XLexAop2-CsChrimson-tdTomato (VK00005) / 70B03-ZpGAL4DBD (attP2)*	*SS58002*
Fig. 4b and Supplementary Fig. 3b	*w / w, UAS-TNTe, VT049484-ZpLexADBD (JK22c) / VT036874-p65ADZp (attP40); VT050660-p65ADZp (attP2), 13XLexAop2-CsChrimson-tdTomato (VK00005) / 70B03-ZpGAL4DBD (attP2)*	*SS58005*
Fig. 4b and Supplementary Fig. 3b	*w / w, UAS-TNTe, VT049484-ZpLexADBD (JK22c) / VT019902-p65ADZp (attP40); VT050660-p65ADZp (attP2), 13XLexAop2-CsChrimson-tdTomato (VK00005) / 72D01-ZpGAL4DBD (attP2)*	*SS58006*
Fig. 4b and Supplementary Fig. 3b	*w / w, UAS-TNTe, VT049484-ZpLexADBD (JK22c) / VT041421-p65ADZp (attP40); VT050660-p65ADZp (attP2), 13XLexAop2-CsChrimson-tdTomato (VK00005) / VT000254-ZpGAL4DBD (attP2)*	*SS59209*
Fig. 4b and Supplementary Fig. 3b	*w / w, UAS-TNTe, VT049484-ZpLexADBD (JK22c) / VT006545-p65ADZp (attP40); VT050660-p65ADZp (attP2), 13XLexAop2-CsChrimson-tdTomato (VK00005) / 29G03-ZpGAL4DBD (attP2)*	*SS59225*
Fig. 4b and Supplementary Fig. 3b	*w / w, UAS-TNTe, VT049484-ZpLexADBD (JK22c) / VT033282-p65ADZp (attP40); VT050660-p65ADZp (attP2), 13XLexAop2-CsChrimson-tdTomato (VK00005) / 75H07-ZpGAL4DBD (attP2)*	*SS59229*
Fig. 4b and Supplementary Fig. 3b	*w / w, UAS-TNTe, VT049484-ZpLexADBD (JK22c) / VT012719-p65ADZp (attP40); VT050660-p65ADZp (attP2), 13XLexAop2-CsChrimson-tdTomato (VK00005) / 75H07-ZpGAL4DBD (attP2)*	*SS59230*
Fig. 4b and Supplementary Fig. 3b	*w / w, UAS-TNTe, VT049484-ZpLexADBD (JK22c) / VT006555-p65ADZp (attP40); VT050660-p65ADZp (attP2), 13XLexAop2-CsChrimson-tdTomato (VK00005) / VT002075-ZpGAL4DBD (attP2)*	*SS60232*
Fig. 4b and Supplementary Fig. 3b	*w / w, UAS-TNTe, VT049484-ZpLexADBD (JK22c) / VT006555-p65ADZp (attP40); VT050660-p65ADZp (attP2), 13XLexAop2-CsChrimson-tdTomato (VK00005) / VT050105-ZpGAL4DBD (attP2)*	*SS60234*
Fig. 4b and Supplementary Fig. 3b	*w / w, UAS-TNTe, VT049484-ZpLexADBD (JK22c) / VT001497-p65ADZp (attP40); VT050660-p65ADZp (attP2), 13XLexAop2-CsChrimson-tdTomato (VK00005) / VT049903-ZpGAL4DBD (attP2)*	*SS60585*
Fig. 4b and Supplementary Fig. 3b	*w / w, UAS-TNTe, VT049484-ZpLexADBD (JK22c) / VT026993-p65ADZp (attP40); VT050660-p65ADZp (attP2), 13XLexAop2-CsChrimson-tdTomato (VK00005) / VT044492-ZpGAL4DBD (attP2)*	*SS62148*
Fig. 4b and Supplementary Fig. 3b	*w / w, UAS-TNTe, VT049484-ZpLexADBD (JK22c) / VT002223-p65ADZp (attP40); VT050660-p65ADZp (attP2), 13XLexAop2-CsChrimson-tdTomato (VK00005) / VT004419-ZpGAL4DBD (attP2)*	*SS62883*
Fig. 4b and Supplementary Fig. 3b	*w / w, UAS-TNTe, VT049484-ZpLexADBD (JK22c) / VT019307-p65ADZp (attP40); VT050660-p65ADZp (attP2), 13XLexAop2-CsChrimson-tdTomato (VK00005) / 50C03-ZpGAL4DBD (attP2)*	*SS63843*
Fig. 4b and Supplementary Fig. 3b	*w / w, UAS-TNTe, VT049484-ZpLexADBD (JK22c) / VT002223-p65ADZp (attP40); VT050660-p65ADZp (attP2), 13XLexAop2-CsChrimson-tdTomato (VK00005) / 21E02-ZpGAL4DBD (attP2)*	*SS64165*
Fig. 4b and Supplementary Fig. 3b	*w / w, UAS-TNTe, VT049484-ZpLexADBD (JK22c) / 78A04-p65ADZp (attP40); VT050660-p65ADZp (attP2), 13XLexAop2-CsChrimson-tdTomato (VK00005) / 24B11-ZpGAL4DBD (attP2)*	*SS64167*
Fig. 4b and Supplementary Fig. 3b	*w / w, UAS-TNTe, VT049484-ZpLexADBD (JK22c) / VT044843-p65ADZp (attP40); VT050660-p65ADZp (attP2), 13XLexAop2-CsChrimson-tdTomato (VK00005) / VT008808-ZpGAL4DBD (attP2)*	*SS64172*
Fig. 4b and Supplementary Fig. 3b	*w / w, UAS-TNTe, VT049484-ZpLexADBD (JK22c) / VT056808-p65ADZp (attP40); VT050660-p65ADZp (attP2), 13XLexAop2-CsChrimson-tdTomato (VK00005) / 24B11-ZpGAL4DBD (attP2)*	*SS64179*
Fig. 4b and Supplementary Fig. 3b	*w / w, UAS-TNTe, VT049484-ZpLexADBD (JK22c) / VT058545-p65ADZp (attP40); VT050660-p65ADZp (attP2), 13XLexAop2-CsChrimson-tdTomato (VK00005) / VT016278-ZpGAL4DBD (attP2)*	*SS64188*

Fig. 4b and Supplementary Fig. 3b	*w / w, UAS-TNTe, VT049484-ZpLexADBD (JK22c) / VT009674-p65ADZp (attP40); VT050660-p65ADZp (attP2), 13XLexAop2-CsChrimson-tdTomato (VK00005) / VT021842-ZpGAL4DBD (attP2)*	*SS64190*
Fig. 4b and Supplementary Fig. 3b	*w / w, UAS-TNTe, VT049484-ZpLexADBD (JK22c) / 24B11-p65ADZp (attP40); VT050660-p65ADZp (attP2), 13XLexAop2-CsChrimson-tdTomato (VK00005) / 78A04-ZpGAL4DBD (attP2)*	*SS64214*
Fig. 4b and Supplementary Fig. 3b	*w / w, UAS-TNTe, VT049484-ZpLexADBD (JK22c) / 19D12-p65ADZp (attP40); VT050660-p65ADZp (attP2), 13XLexAop2-CsChrimson-tdTomato (VK00005) / VT058545-ZpGAL4DBD (attP2)*	*SS64225*
Fig. 4b and Supplementary Fig. 3b	*w / w, UAS-TNTe, VT049484-ZpLexADBD (JK22c) / VT008808-p65ADZp (attP40); VT050660-p65ADZp (attP2), 13XLexAop2-CsChrimson-tdTomato (VK00005) / VT002042-ZpGAL4DBD (attP2)*	*SS64234*
Fig. 4b and Supplementary Fig. 3b	*w / w, UAS-TNTe, VT049484-ZpLexADBD (JK22c) / VT024647-p65ADZp (attP40); VT050660-p65ADZp (attP2), 13XLexAop2-CsChrimson-tdTomato (VK00005) / VT049344-ZpGAL4DBD (attP2)*	*SS65706*
Fig. 4b and Supplementary Fig. 3b	*w / w, UAS-TNTe, VT049484-ZpLexADBD (JK22c) / VT045636-p65ADZp (attP40); VT050660-p65ADZp (attP2), 13XLexAop2-CsChrimson-tdTomato (VK00005) / VT024647-ZpGAL4DBD (attP2)*	*SS65713*
Fig. 4b and Supplementary Fig. 3b	*w / w, UAS-TNTe, VT049484-ZpLexADBD (JK22c) / 68A10-p65ADZp (attP40); VT050660-p65ADZp (attP2), 13XLexAop2-CsChrimson-tdTomato (VK00005) / 53C10-ZpGAL4DBD (attP2)*	*SS66965*
Fig. 4b and Supplementary Fig. 3b	*w / w, UAS-TNTe, VT049484-ZpLexADBD (JK22c) / VT014336-p65ADZp (attP40); VT050660-p65ADZp (attP2), 13XLexAop2-CsChrimson-tdTomato (VK00005) / 20F06-ZpGAL4DBD (attP2)*	*SS67323*
Fig. 4b and Supplementary Fig. 3b	*w / w, UAS-TNTe, VT049484-ZpLexADBD (JK22c) / VT014336-p65ADZp (attP40); VT050660-p65ADZp (attP2), 13XLexAop2-CsChrimson-tdTomato (VK00005) / 81A06-ZpGAL4DBD (attP2)*	*SS67324*
Fig. 4b and Supplementary Fig. 3b	*w / w, UAS-TNTe, VT049484-ZpLexADBD (JK22c) / VT040713-p65ADZp (attP40); VT050660-p65ADZp (attP2), 13XLexAop2-CsChrimson-tdTomato (VK00005) / VT045636-ZpGAL4DBD (attP2)*	*SS67354*
Fig. 4b and Supplementary Fig. 3b	*w / w, UAS-TNTe, VT049484-ZpLexADBD (JK22c) / 20F06-p65ADZp (attP40); VT050660-p65ADZp (attP2), 13XLexAop2-CsChrimson-tdTomato (VK00005) / 81A06-ZpGAL4DBD (attP2)*	*SS67423*
Fig. 4b and Supplementary Fig. 3b	*w / w, UAS-TNTe, VT049484-ZpLexADBD (JK22c) / 59F08-p65ADZp (attP40); VT050660-p65ADZp (attP2), 13XLexAop2-CsChrimson-tdTomato (VK00005) / 45E06-ZpGAL4DBD (attP2)*	*SS67463*
Fig. 4b and Supplementary Fig. 3b	*w / w, UAS-TNTe, VT049484-ZpLexADBD (JK22c) / 65E11-p65ADZp (attP40); VT050660-p65ADZp (attP2), 13XLexAop2-CsChrimson-tdTomato (VK00005) / VT000353-ZpGAL4DBD (attP2)*	*SS67466*
Fig. 4b and Supplementary Fig. 3b	*w / w, UAS-TNTe, VT049484-ZpLexADBD (JK22c) / 82F02-p65ADZp (attP40); VT050660-p65ADZp (attP2), 13XLexAop2-CsChrimson-tdTomato (VK00005) / VT049125-ZpGAL4DBD (attP2)*	*SS67472*
Fig. 4b and Supplementary Fig. 3b	*w / w, UAS-TNTe, VT049484-ZpLexADBD (JK22c) / VT001608-p65ADZp (attP40); VT050660-p65ADZp (attP2), 13XLexAop2-CsChrimson-tdTomato (VK00005) / VT014554-ZpGAL4DBD (attP2)*	*SS67878*
Fig. 4b and Supplementary Fig. 3b	*w / w, UAS-TNTe, VT049484-ZpLexADBD (JK22c) / VT039419-p65ADZp (attP40); VT050660-p65ADZp (attP2), 13XLexAop2-CsChrimson-tdTomato (VK00005) / VT014974-ZpGAL4DBD (attP2)*	*SS67907*
Supplementary Fig. 3a	*20XUAS-CsChrimson-mVenus (attP18), w / w; VT039419-p65ADZp (attP40) / +; VT014974-ZpGAL4DBD (attP2) / +*	*SS27416*
Supplementary Fig. 3a	*20XUAS-CsChrimson-mVenus (attP18), w / w; 72E10-p65ADZp (attP40) / +; VT049904-ZpGAL4DBD (attP2) / +*	*SS32496*
Supplementary Fig. 3a	*20XUAS-CsChrimson-mVenus (attP18), w / w; VT043924-p65ADZp (attP40) / +; VT044964-ZpGAL4DBD (attP2) / +*	*SS33343*
Supplementary Fig. 3a	*20XUAS-CsChrimson-mVenus (attP18), w / w; VT033629-p65ADZp (attP40) / +; VT021799-ZpGAL4DBD (attP2) / +*	*SS33510*
Supplementary Fig. 3a	*20XUAS-CsChrimson-mVenus (attP18), w / w; VT044964-p65ADZp (attP40) / +; VT023795-ZpGAL4DBD (attP2) / +*	*SS33542*
Supplementary Fig. 3a	*20XUAS-CsChrimson-mVenus (attP18), w / w; 77B09-p65ADZp (attP40) / +; 72c08-ZpGAL4DBD (attP2) / +*	*SS35560*

Supplementary Fig. 3a	*20XUAS-CsChrimson-mVenus (attP18), w / w; 92A10-p65ADZp (attP40) / +; VT019902-ZpGAL4DBD (attP2) / +*	*SS36194*
Supplementary Fig. 3a	*20XUAS-CsChrimson-mVenus (attP18), w / w; VT001608-p65ADZp (attP40) / +; VT025916-ZpGAL4DBD (attP2) / +*	*SS38205*
Supplementary Fig. 3a	*20XUAS-CsChrimson-mVenus (attP18), w / w; VT063303-p65ADZp (attP40) / +; VT063741-ZpGAL4DBD (attP2) / +*	*SS39036*
Supplementary Fig. 3a	*20XUAS-CsChrimson-mVenus (attP18), w / w; 78A01-p65ADZp (attP40) / +; VT009084-ZpGAL4DBD (attP2) / +*	*SS43697*
Supplementary Fig. 3a	*20XUAS-CsChrimson-mVenus (attP18), w / w; VT064563-p65ADZp (attP40) / +; VT000254-ZpGAL4DBD (attP2) / +*	*SS44225*
Supplementary Fig. 3a	*20XUAS-CsChrimson-mVenus (attP18), w / w; VT065322-p65ADZp (attP40) / +; VT019902-ZpGAL4DBD (attP2) / +*	*SS44254*
Supplementary Fig. 3a	*20XUAS-CsChrimson-mVenus (attP18), w / w; VT065322-p65ADZp (attP40) / +; 92A10-ZpGAL4DBD (attP2) / +*	*SS44255*
Supplementary Fig. 3a	*20XUAS-CsChrimson-mVenus (attP18), w / w; VT005404-p65ADZp (attP40) / +; VT000254-ZpGAL4DBD (attP2) / +*	*SS46225*
Supplementary Fig. 3a	*20XUAS-CsChrimson-mVenus (attP18), w / w; VT033616-p65ADZp (attP40) / +; VT008473-ZpGAL4DBD (attP2) / +*	*SS46229*
Supplementary Fig. 3a	*20XUAS-CsChrimson-mVenus (attP18), w / w; VT029814-p65ADZp (attP40) / +; VT028464-ZpGAL4DBD (attP2) / +*	*SS46233*
Supplementary Fig. 3a	*20XUAS-CsChrimson-mVenus (attP18), w / w; VT006480-p65ADZp (attP40) / +; 24A02-ZpGAL4DBD (attP2) / +*	*SS46243*
Supplementary Fig. 3a	*20XUAS-CsChrimson-mVenus (attP18), w / w; 24A02-p65ADZp (attP40) / +; VT006480-ZpGAL4DBD (attP2) / +*	*SS46253*
Supplementary Fig. 3a	*20XUAS-CsChrimson-mVenus (attP18), w / w; 31H09-p65ADZp (attP40) / +; VT020829-ZpGAL4DBD (attP2) / +*	*SS46258*
Supplementary Fig. 3a	*20XUAS-CsChrimson-mVenus (attP18), w / w; VT043146-p65ADZp (attP40) / +; VT000254-ZpGAL4DBD (attP2) / +*	*SS46300*
Supplementary Fig. 3a	*20XUAS-CsChrimson-mVenus (attP18), w / w; VT026777-p65ADZp (attP40) / +; VT049125-ZpGAL4DBD (attP2) / +*	*SS46669*
Supplementary Fig. 3a	*20XUAS-CsChrimson-mVenus (attP18), w / w; VT037566-p65ADZp (attP40) / +; VT008808-ZpGAL4DBD (attP2) / +*	*SS46730*
Supplementary Fig. 3a	*20XUAS-CsChrimson-mVenus (attP18), w / w; VT058873-p65ADZp (attP40) / +; VT058566-ZpGAL4DBD (attP2) / +*	*SS47847*
Supplementary Fig. 3a	*20XUAS-CsChrimson-mVenus (attP18), w / w; 15D08-p65ADZp (attP40) / +; 72E10-ZpGAL4DBD (attP2) / +*	*SS47863*
Supplementary Fig. 3a	*20XUAS-CsChrimson-mVenus (attP18), w / w; VT021418-p65ADZp (attP40) / +; 24A02-ZpGAL4DBD (attP2) / +*	*SS47921*
Supplementary Fig. 3a	*20XUAS-CsChrimson-mVenus (attP18), w / w; 24A02-p65ADZp (attP40) / +; VT021418-ZpGAL4DBD (attP2) / +*	*SS47938*
Supplementary Fig. 3a	*20XUAS-CsChrimson-mVenus (attP18), w / w; VT008808-p65ADZp (attP40) / +; VT048646-ZpGAL4DBD (attP2) / +*	*SS48376*
Supplementary Fig. 3a	*20XUAS-CsChrimson-mVenus (attP18), w / w; 75B10-p65ADZp (attP40) / +; 94E11-ZpGAL4DBD (attP2) / +*	*SS48718*
Supplementary Fig. 3a	*20XUAS-CsChrimson-mVenus (attP18), w / w; VT062245-p65ADZp (attP40) / +; VT050105-ZpGAL4DBD (attP2) / +*	*SS50974*
Supplementary Fig. 3a	*20XUAS-CsChrimson-mVenus (attP18), w / w; VT062245-p65ADZp (attP40) / +; VT021374-ZpGAL4DBD (attP2) / +*	*SS50975*
Supplementary Fig. 3a	*20XUAS-CsChrimson-mVenus (attP18), w / w; 24A02-p65ADZp (attP40) / +; VT000360-ZpGAL4DBD (attP2) / +*	*SS50995*
Supplementary Fig. 3a	*20XUAS-CsChrimson-mVenus (attP18), w / w; 31H05-p65ADZp (attP40) / +; VT011123-ZpGAL4DBD (attP2) / +*	*SS50996*
Supplementary Fig. 3a	*20XUAS-CsChrimson-mVenus (attP18), w / w; VT024602-p65ADZp (attP40) / +; VT021374-ZpGAL4DBD (attP2) / +*	*SS51817*
Supplementary Fig. 3a	*20XUAS-CsChrimson-mVenus (attP18), w / w; VT021418-p65ADZp (attP40) / +; VT012717-ZpGAL4DBD (attP2) / +*	*SS51843*
Supplementary Fig. 3a	*20XUAS-CsChrimson-mVenus (attP18), w / w; 23G01-p65ADZp (attP40) / +; VT037810-ZpGAL4DBD (attP2) / +*	*SS51877*
Supplementary Fig. 3a	*20XUAS-CsChrimson-mVenus (attP18), w / w; 28F07-p65ADZp (attP40) / +; VT008808-ZpGAL4DBD (attP2) / +*	*SS51891*

Supplementary Fig. 3a	*20XUAS-CsChrimson-mVenus (attP18), w / w; 47A08-p65ADZp (attP40) / +; VT029814-ZpGAL4DBD (attP2) / +*	*SS51895*
Supplementary Fig. 3a	*20XUAS-CsChrimson-mVenus (attP18), w / w; 70E04-p65ADZp (attP40) / +; VT021418-ZpGAL4DBD (attP2) / +*	*SS51907*
Supplementary Fig. 3a	*20XUAS-CsChrimson-mVenus (attP18), w / w; 24E12-p65ADZp (attP40) / +; VT019345-ZpGAL4DBD (attP2) / +*	*SS53029*
Supplementary Fig. 3a	*20XUAS-CsChrimson-mVenus (attP18), w / w; 24E12-p65ADZp (attP40) / +; VT048146-ZpGAL4DBD (attP2) / +*	*SS53030*
Supplementary Fig. 3a	*20XUAS-CsChrimson-mVenus (attP18), w / w; 81A04-p65ADZp (attP40) / +; 81A06-ZpGAL4DBD (attP2) / +*	*SS53050*
Supplementary Fig. 3a	*20XUAS-CsChrimson-mVenus (attP18), w / w; VT002081-p65ADZp (attP40) / +; VT019345-ZpGAL4DBD (attP2) / +*	*SS53903*
Supplementary Fig. 3a	*20XUAS-CsChrimson-mVenus (attP18), w / w; VT029814-p65ADZp (attP40) / +; VT049340-ZpGAL4DBD (attP2) / +*	*SS57939*
Supplementary Fig. 3a	*20XUAS-CsChrimson-mVenus (attP18), w / w; VT049362-p65ADZp (attP40) / +; VT021374-ZpGAL4DBD (attP2) / +*	*SS57941*
Supplementary Fig. 3a	*20XUAS-CsChrimson-mVenus (attP18), w / w; VT029814-p65ADZp (attP40) / +; VT025717-ZpGAL4DBD (attP2) / +*	*SS57954*
Supplementary Fig. 3a	*20XUAS-CsChrimson-mVenus (attP18), w / w; VT019307-p65ADZp (attP40) / +; VT025717-ZpGAL4DBD (attP2) / +*	*SS57955*
Supplementary Fig. 3a	*20XUAS-CsChrimson-mVenus (attP18), w / w; VT062633-p65ADZp (attP40) / +; VT008808-ZpGAL4DBD (attP2) / +*	*SS57977*
Supplementary Fig. 3a	*20XUAS-CsChrimson-mVenus (attP18), w / w; VT062633-p65ADZp (attP40) / +; 28F07-ZpGAL4DBD (attP2) / +*	*SS58000*
Supplementary Fig. 3a	*20XUAS-CsChrimson-mVenus (attP18), w / w; VT045608-p65ADZp (attP40) / +; 70B03-ZpGAL4DBD (attP2) / +*	*SS58002*
Supplementary Fig. 3a	*20XUAS-CsChrimson-mVenus (attP18), w / w; VT036874-p65ADZp (attP40) / +; 70B03-ZpGAL4DBD (attP2) / +*	*SS58005*
Supplementary Fig. 3a	*20XUAS-CsChrimson-mVenus (attP18), w / w; VT019902-p65ADZp (attP40) / +; 72D01-ZpGAL4DBD (attP2) / +*	*SS58006*
Supplementary Fig. 3a	*20XUAS-CsChrimson-mVenus (attP18), w / w; VT041421-p65ADZp (attP40) / +; VT000254-ZpGAL4DBD (attP2) / +*	*SS59209*
Supplementary Fig. 3a	*20XUAS-CsChrimson-mVenus (attP18), w / w; VT006545-p65ADZp (attP40) / +; 29G03-ZpGAL4DBD (attP2) / +*	*SS59225*
Supplementary Fig. 3a	*20XUAS-CsChrimson-mVenus (attP18), w / w; VT033282-p65ADZp (attP40) / +; 75H07-ZpGAL4DBD (attP2) / +*	*SS59229*
Supplementary Fig. 3a	*20XUAS-CsChrimson-mVenus (attP18), w / w; VT012719-p65ADZp (attP40) / +; 75H07-ZpGAL4DBD (attP2) / +*	*SS59230*
Supplementary Fig. 3a	*20XUAS-CsChrimson-mVenus (attP18), w / w; VT006555-p65ADZp (attP40) / +; VT002075-ZpGAL4DBD (attP2) / +*	*SS60232*
Supplementary Fig. 3a	*20XUAS-CsChrimson-mVenus (attP18), w / w; VT006555-p65ADZp (attP40) / +; VT050105-ZpGAL4DBD (attP2) / +*	*SS60234*
Supplementary Fig. 3a	*20XUAS-CsChrimson-mVenus (attP18), w / w; VT001497-p65ADZp (attP40) / +; VT049903-ZpGAL4DBD (attP2) / +*	*SS60585*
Supplementary Fig. 3a	*20XUAS-CsChrimson-mVenus (attP18), w / w; VT026993-p65ADZp (attP40) / +; VT044492-ZpGAL4DBD (attP2) / +*	*SS62148*
Supplementary Fig. 3a	*20XUAS-CsChrimson-mVenus (attP18), w / w; VT002223-p65ADZp (attP40) / +; VT004419-ZpGAL4DBD (attP2) / +*	*SS62883*
Supplementary Fig. 3a	*20XUAS-CsChrimson-mVenus (attP18), w / w; VT019307-p65ADZp (attP40) / +; 50C03-ZpGAL4DBD (attP2) / +*	*SS63843*
Supplementary Fig. 3a	*20XUAS-CsChrimson-mVenus (attP18), w / w; VT002223-p65ADZp (attP40) / +; 21E02-ZpGAL4DBD (attP2) / +*	*SS64165*
Supplementary Fig. 3a	*20XUAS-CsChrimson-mVenus (attP18), w / w; 78A04-p65ADZp (attP40) / +; 24B11-ZpGAL4DBD (attP2) / +*	*SS64167*
Supplementary Fig. 3a	*20XUAS-CsChrimson-mVenus (attP18), w / w; VT044843-p65ADZp (attP40) / +; VT008808-ZpGAL4DBD (attP2) / +*	*SS64172*
Supplementary Fig. 3a	*20XUAS-CsChrimson-mVenus (attP18), w / w; VT056808-p65ADZp (attP40) / +; 24B11-ZpGAL4DBD (attP2) / +*	*SS64179*
Supplementary Fig. 3a	*20XUAS-CsChrimson-mVenus (attP18), w / w; VT058545-p65ADZp (attP40) / +; VT016278-ZpGAL4DBD (attP2) / +*	*SS64188*

Supplementary Fig. 3a	20XUAS-CsChrimson-mVenus (attP18), w / w; VT009674-p65ADZp (attP40) / +; VT021842-ZpGAL4DBD (attP2) / +	SS64190
Supplementary Fig. 3a	20XUAS-CsChrimson-mVenus (attP18), w / w; 24B11-p65ADZp (attP40) / +; 78A04-ZpGAL4DBD (attP2) / +	SS64214
Supplementary Fig. 3a	20XUAS-CsChrimson-mVenus (attP18), w / w; 19D12-p65ADZp (attP40) / +; VT058545-ZpGAL4DBD (attP2) / +	SS64225
Supplementary Fig. 3a	20XUAS-CsChrimson-mVenus (attP18), w / w; VT008808-p65ADZp (attP40) / +; VT002042-ZpGAL4DBD (attP2) / +	SS64234
Supplementary Fig. 3a	20XUAS-CsChrimson-mVenus (attP18), w / w; VT024647-p65ADZp (attP40) / +; VT049344-ZpGAL4DBD (attP2) / +	SS65706
Supplementary Fig. 3a	20XUAS-CsChrimson-mVenus (attP18), w / w; VT045636-p65ADZp (attP40) / +; VT024647-ZpGAL4DBD (attP2) / +	SS65713
Supplementary Fig. 3a	20XUAS-CsChrimson-mVenus (attP18), w / w; 68A10-p65ADZp (attP40) / +; 53C10-ZpGAL4DBD (attP2) / +	SS66965
Supplementary Fig. 3a	20XUAS-CsChrimson-mVenus (attP18), w / w; VT014336-p65ADZp (attP40) / +; 20F06-ZpGAL4DBD (attP2) / +	SS67323
Supplementary Fig. 3a	20XUAS-CsChrimson-mVenus (attP18), w / w; VT014336-p65ADZp (attP40) / +; 81A06-ZpGAL4DBD (attP2) / +	SS67324
Supplementary Fig. 3a	20XUAS-CsChrimson-mVenus (attP18), w / w; VT040713-p65ADZp (attP40) / +; VT045636-ZpGAL4DBD (attP2) / +	SS67354
Supplementary Fig. 3a	20XUAS-CsChrimson-mVenus (attP18), w / w; 20F06-p65ADZp (attP40) / +; 81A06-ZpGAL4DBD (attP2) / +	SS67423
Supplementary Fig. 3a	20XUAS-CsChrimson-mVenus (attP18), w / w; 59F08-p65ADZp (attP40) / +; 45E06-ZpGAL4DBD (attP2) / +	SS67463
Supplementary Fig. 3a	20XUAS-CsChrimson-mVenus (attP18), w / w; 65E11-p65ADZp (attP40) / +; VT000353-ZpGAL4DBD (attP2) / +	SS67466
Supplementary Fig. 3a	20XUAS-CsChrimson-mVenus (attP18), w / w; 82F02-p65ADZp (attP40) / +; VT049125-ZpGAL4DBD (attP2) / +	SS67472
Supplementary Fig. 3a	20XUAS-CsChrimson-mVenus (attP18), w / w; VT001608-p65ADZp (attP40) / +; VT014554-ZpGAL4DBD (attP2) / +	SS67878
Supplementary Fig. 3a	20XUAS-CsChrimson-mVenus (attP18), w / w; VT039419-p65ADZp (attP40) / +; VT014974-ZpGAL4DBD (attP2) / +	SS67907
Supplementary Fig. 4A	w / w, UAS-TNTe, VT049484-ZpLexADBD (JK22c) / 24A02-p65ADZp (attP40) ;VT050660-p65ADZp (attP2), 13XLexAop2-CsChrimson-tdTomato (VK00005) / VT021418-ZpGAL4DBD (attP2)	MDN>Chrimson-tdTomato SS47938>TNT
Supplementary Fig. 4B	w / w, UAS-TNTe, VT049484-ZpLexADBD (JK22c) / VT062245-p65ADZp (attP40) ;VT050660-p65ADZp (attP2), 13XLexAop2-CsChrimson-tdTomato (VK00005) / VT050105-ZpGAL4DBD (attP2)	MDN>Chrimson-tdTomato SS50974>TNT
Fig. 5a, 6a and Supplementary Fig. 6e	pJFRC300-20XUAS-FRT-dSTOP-FRTCsChrimson::mVenus (attP18), hs-FLP-PESTOpt (attP3), w / w; VT044845-ZpGAL4DBD (attP40) / +; VT050660-p65ADZp (attP2) / +	MDN
Fig. 5a,h	R57C10-Flp2::PEST (attp18), w / w; brp::Snap / +; pJFRC201-10XUAS-FRT>STOP>FRT-myr::smGFP-HA (VK00005), pJFRC240-10XUAS-FRT>STOP>FRT-myr::smGFP-V5-THS-10XUAS-FRT>STOP>FRT-myr::smGFP-FLAG (su(Hw)attP1) / VT029570-GAL4 (attP2)	LBL40
Fig. 5b	20XUAS-CsChrimson-mVenus (attP18), w / w; 24A02-p65ADZp (attP40) / +; VT021418-ZpGAL4DBD (attP2) / +	SS47938>mVenus
Fig. 5b	w / w, VT044845-LexAp65 (attp40) / 24A02-p65ADZp (attP40); 13XLexAop2-CsChrimson-tdTomato (VK00005), 20XUAS Syn21-opGCaMP6s-p10-bp (su(Hw)attP1) / VT021418-ZpGAL4DBD (attP2)	MDN>CsChrimson SS47938>GCaMP6s
Fig. 5b	20XUAS-CsChrimson-mVenus (attP18), w / w; VT006480-p65ADZp (attP40) / +; 24A02-ZpGAL4DBD (attP2) / +	SS46243>mVenus
Fig. 5b	w / w, VT044845-LexAp65 (attp40) / VT006480-p65ADZp (attP40); 13XLexAop2-CsChrimson-tdTomato (VK00005), 20XUAS Syn21-opGCaMP6s-p10-bp (su(Hw)attP1) / 24A02-ZpGAL4DBD (attP2)	MDN>CsChrimson SS46243>GCaMP6s
Fig. 5c-g and Supplementary Fig. 6c,d	20XUAS-CsChrimson-mVenus (attP18), w / w; 24A02-p65ADZp (attP40) / +; VT021418-ZpGAL4DBD (attP2) / +	SS47938>CsChrimson
Fig. 5d,e and Supplementary Fig. 6c,d	20XUAS-CsChrimson-mVenus (attP18), w / w; VT006480-p65ADZp (attP40) / +; 24A02-ZpGAL4DBD (attP2) / +	SS46243>CsChrimson

Fig. 5d,e and Supplementary Fig. 6c,d	20XUAS-CsChrimson-mVenus (attP18), w / w; pBPp65ADZpUw* (attP40) / +; pBPZpGAL4DBDUw* (attP2) / +	Empty-SS>CsChrimson
Fig. 5h and Supplementary Fig. 6e	R57C10-Flp2::PEST (attp18), w / w; 31H05-p65ADZp (attp40) / +; VT011123-ZpGAL4DBD (attp2), pJFRC201-10XUAS-FRT>STOP>FRT-myr::smGFP-HA (VK00005), pJFRC240-10XUAS-FRT>STOP>FRT-myr::smGFP-V5-THS-10XUAS-FRT>STOP>FRT-myr::smGFP-FLAG (su(Hw)attP1)	LUM9
Fig. 5i	w / w; VT021418-LexAGADfl (attP40) / +; 13XLexAop2-IVS-myr::GFP (attP2) / +	VT021418>GFP
Fig. 5i	20XUAS-CsChrimson-mVenus (attP18), w / w; 31H05-p65ADZp (attP40) / +; VT011123-ZpGAL4DBD (attP2) / +	SS50996>mVenus
Fig. 5i	w / w; VT021418-LexAGADfl (attP40) / 31H05-p65ADZp (attP40); 13XLexAop2-CsChrimson-tdTomato (VK00005), 20XUAS Syn21-opGCaMP6s-p10-bp (su(Hw)attP1) / VT011123-ZpGAL4DBD (attP2)	VT021418>CsChrimson VT050996>GCaMP6s
Supplementary Fig. 6a	20XUAS-CsChrimson-mVenus (attP18), w / yw; 31H05-p65ADZp (attP40) / Mhc-RFP; VT011123-ZpGAL4DBD (attP2) / +	SS50996>mVenus Mhc-RFP
Supplementary Fig. 6b-d	20XUAS-CsChrimson-mVenus (attP18), w / w; 31H05-p65ADZp (attP40) / +; VT011123-ZpGAL4DBD (attP2) / +	SS50996>CsChrimson
Supplementary Fig. 6e	LexAop2-Syn21-opGCamp6s (su(HW)8), 10XUAS Syn21-Chrimson88-tdt3.1 (attP18), w / w; VT044845-ZpGAL4DBD (attP40) / 31H05-p65ADZp (attP40); VT050660-p65ADZp (attP2) / VT011123-ZpGAL4DBD (attP2)	MDN>CsChrimson SS50996>GCaMP6s
Supplementary Fig. 6f	w / w; VT021418-LexAGADfl (attP40), pJFRC48-13XLexAop2-IVS-myrtdTomato (su(Hw)attP5) / 31H05-p65ADZp (attP40); P{w[+mC]=UAS-CD4-spGFP1-10}3, P{w[+mC]=lexAop-CD4-spGFP11}3 / VT011123-ZpGAL4DBD (attP2)	LBL40>tdT, GFP11 LUM9>GFP1-10
Supplementary Fig. 6f	w / w; VT021418-LexAGADfl (attP40), pJFRC48-13XLexAop2-IVS-myrtdTomato (su(Hw)attP5) / +; P{w[+mC]=UAS-CD4-spGFP1-10}3, P{w[+mC]=lexAop-CD4-spGFP11}3 / +	LBL40>tdT, GFP11 UAS-GFP1-10
Supplementary Fig. 6f	w / w; pJFRC48-13XLexAop2-IVS-myrtdTomato (su(Hw)attP5) / 31H05-p65ADZp (attP40); P{w[+mC]=UAS-CD4-spGFP1-10}3, P{w[+mC]=lexAop-CD4-spGFP11}3 / VT011123-ZpGAL4DBD (attP2)	LexAop-tdT, GFP11 LUM9>GFP1-10
Fig. 6a	R57C10-Flp2::PEST (attp18), w / w; brp::Snap / +; pJFRC201-10XUAS-FRT>STOP>FRT-myr::smGFP-HA (VK00005), pJFRC240-10XUAS-FRT>STOP>FRT-myr::smGFP-V5-THS-10XUAS-FRT>STOP>FRT-myr::smGFP-FLAG (su(Hw)attP1) / VT062245-GAL4 (attP2)	LUL130
Fig. 6b	20XUAS-CsChrimson-mVenus (attP18), w / w; VT062245-p65ADZp (attP40) / +; VT050105-ZpGAL4DBD (attP2) / +	SS50974>mVenus
Fig. 6b	w / w, VT044845-LexAp65 (attp40) / VT062245-p65ADZp (attP40); 13XLexAop2-CsChrimson-tdTomato (VK00005), 20XUAS Syn21-opGCaMP6s-p10-bp (su(Hw)attP1) / VT050105-ZpGAL4DBD (attP2)	MDN>CsChrimson SS50974>GCaMP6s
Fig. 6b	20XUAS-CsChrimson-mVenus (attP18), w / w; VT062245-p65ADZp (attP40) / +; VT021374-ZpGAL4DBD (attP2) / +	SS50975>mVenus
Fig. 6b	w / w, VT044845-LexAp65 (attp40) / VT062245-p65ADZp (attP40); 13XLexAop2-CsChrimson-tdTomato (VK00005), 20XUAS Syn21-opGCaMP6s-p10-bp (su(Hw)attP1) / VT021374-ZpGAL4DBD (attP2)	MDN>CsChrimson SS50975>GCaMP6s
Fig. 6c-f	20XUAS-CsChrimson-mVenus (attP18), w / w; VT062245-p65ADZp (attP40) / +; VT050105-ZpGAL4DBD (attP2) / +	SS50974>CsChrimson
Fig. 6d,e,g,h	20XUAS-CsChrimson-mVenus (attP18), w / w; VT062245-p65ADZp (attP40) / +; VT021374-ZpGAL4DBD (attP2) / +	SS50975>CsChrimson
Fig. 6d,e	20XUAS-CsChrimson-mVenus (attP18), w / w; pBPp65ADZpUw* (attP40) / +; pBPZpGAL4DBDUw* (attP2) / +	Empty-SS>CsChrimson
Fig. 7b	13XLexAop2-CsChrimson-tdTomato (attP18), w / w; VT049484-ZpLexADBD (JK22c), VT050660-p65ADZp (attP40) / VT044845-ZpGAL4DBD (attP40); UAS-GtACR2 (attP2) / VT050660-p65ADZp (attP2)	MDN>CsChrimson MDN>GtACR2
Fig. 7c-g and Supplementary Fig. 7	13XLexAop2-CsChrimson-tdTomato (attP18), w / w; VT049484-ZpLexADBD (JK22c), VT050660-p65ADZp (attP40) / pBPp65ADZpUw* (attP40); UAS-GtACR2 (attP2) / pBPZpGAL4DBDUw* (attP2)	Empty-SS
Fig. 7c-g and Supplementary Fig. 7	13XLexAop2-CsChrimson-tdTomato (attP18), w / w; VT049484-ZpLexADBD (JK22c), VT050660-p65ADZp (attP40) / 24A02-p65ADZp (attP40); UAS-GtACR2 (attP2) / VT021418-ZpGAL4DBD (attP2)	LBL40: SS47938

Fig. 7c-g and Supplementary Fig. 7	*13XLexAop2-CsChrimson-tdTomato (attP18), w / w; VT049484-ZpLexADBD (JK22c), VT050660-p65ADZp (attP40) / VT062245-p65ADZp (attP40); UAS-GtACR2 (attP2) / VT050105-ZpGAL4DBD (attP2)*	LUL130: *SS50974*

Supplementary Table 2 | Full genotypes of flies used in experiments.

Supplementary References

1 Bidaye, S. S., Machacek, C., Wu, Y. & Dickson, B. J. Neuronal control of *Drosophila* walking direction. *Science* **344**, 97-101 (2014).

2 Sen, R. *et al.* Moonwalker Descending Neurons mediate visually evoked retreat in *Drosophila*. *Curr Biol* **27**, 766-771 (2017).

3 Sen, R., Wang, K. & Dickson, B. J. TwoLumps ascending neurons mediate touch-evoked reversal of walking direction in *Drosophila*. *Curr Biol* **29**, 4337-4344 (2019).

4 Tirian, L. & Dickson, B. J. The VT GAL4, LexA, and split-GAL4 driver line collections for targeted expression in the *Drosophila* nervous system. Preprint at https://www.biorxiv.org/content/10.1101/198648v1 (2017).

5 Jenett, A. *et al.* A GAL4-driver line resource for *Drosophila* neurobiology. *Cell Rep* **2**, 991-1001 (2012).

6 Dionne, H., Hibbard, K. L., Cavallaro, A., Kao, J. C. & Rubin, G. M. Genetic reagents for making split-GAL4 lines in *Drosophila*. *Genetics* **209**, 31-35 (2018).

7 Hampel, S., Franconville, R., Simpson, J. H. & Seeds, A. M. A neural command circuit for grooming movement control. *Elife* **4**, e08758 (2015).

8 Talay, M. *et al.* Transsynaptic mapping of second-order taste neurons in flies by trans-Tango. *Neuron* **96**, 783-795 (2017).

9 Chen, T. W. *et al.* Ultrasensitive fluorescent proteins for imaging neuronal activity. *Nature* **499**, 295-300 (2013).

10 Klapoetke, N. C. *et al.* Independent optical excitation of distinct neural populations. *Nat Methods* **11**, 338-346 (2014).

11 Simpson, J. H. Rationally subdividing the fly nervous system with versatile expression reagents. *J Neurogenet* **30**, 185-194 (2016).

12 Kohl, J. *et al.* Ultrafast tissue staining with chemical tags. *Proc Natl Acad Sci U S A* **111**, E3805-3814 (2014).

13 Nern, A., Pfeiffer, B. D. & Rubin, G. M. Optimized tools for multicolor stochastic labeling reveal diverse stereotyped cell arrangements in the fly visual system. *Proc Natl Acad Sci U S A* **112**, E2967-2976 (2015).

14 Wu, M. *et al.* Visual projection neurons in the *Drosophila* lobula link feature detection to distinct behavioral programs. *Elife* **5**, e21022 (2016).

15 Sweeney, S. T., Broadie, K., Keane, J., Niemann, H. & Okane, C. J. Targeted expression of tetanus toxin light-chain in *Drosophila* specifically eliminates synaptic transmission and causes behavioral defects. *Neuron* **14**, 341-351 (1995).

16 Mohammad, F. *et al.* Optogenetic inhibition of behavior with anion channelrhodopsins. *Nat Methods* **14**, 271-274 (2017).

17 Bosch, J. A., Tran, N. H. & Hariharan, I. K. CoinFLP: a system for efficient mosaic screening and for visualizing clonal boundaries in *Drosophila*. *Development* **142**, 597-606 (2015).

18 Pfeiffer, B. D. *et al.* Refinement of tools for targeted gene expression in *Drosophila*. *Genetics* **186**, 735-755 (2010).

19 Enriquez, J. *et al.* Specification of individual adult motor neuron morphologies by combinatorial transcription factor codes. *Neuron* **86**, 955-970 (2015).
