# Supplementary material for: Distributed control of motor circuits for backward walking in Drosophila
Source: Nat Commun. 2020 Dec 2;11:6166. doi: 10.1038/s41467-020-19936-x (PMC7710706; doi:10.1038/s41467-020-19936-x)
Supplement: Supplementary file 14 — Description of Additional Supplementary Files [file 41467_2020_19936_MOESM14_ESM.docx]

Description of Additional Supplementary Information

Title: Supplementary Movie 1

Description: Joint kinematics during forward and backward walking. A tethered *MDN-1-GAL4>CsChrimson* fly spontaneously walking forward on a ball, then walking backwards upon presentation of a 5-s red-light stimulus (red square). Leg segments (blue) and joints and tips (red) on one side of the body were labeled using DeepLabCut. The trajectory of tarsal tips is plotted in white for forward walking and in red for backward walking. Recorded at 200 fps and rendered at 40 fps.

Title: Supplementary Movie 2

Description: Amputation experiments reveal a dominant role for the hindlegs in backward walking. *MDN-1-GAL4>CsChrimson* flies walking on a ball, before, during, and after a 5-s red-light stimulus (red squares). Flies shown from left to right have the fore-, mid- or hindlegs bilaterally amputated at tarsus, respectively. Recorded at 200 fps and rendered at 40 fps.

Title: Supplementary Movie 3

Description: MDNs preferentially activate T3 neurons. VNC volume of a *MDN-1-GAL4>Chrimson88*, *57C10-LexA>GCaMP6s* fly imaged while MDNs were activated using red light (red squares). Left, maximal-Z projection of raw images averaged across 10 sessions. Right, corresponding of ΔF/F_0_ images with color code indicated at the lower right corner. Recorded at 1 fps and rendered at 5 fps.

Title: Supplementary Movie 4

Description: Chronic neuronal silencing reveals lines with reduced backward walking upon acute MDN activation. Representative walking trajectories of 5 flies in Fly Bowl for each line as indicated in the Movie. The genotypes are *SS>TNT-E,* *VT049484- ZpLexADBD, VT050660-p65ADZp>CsChrimson*. Trajectories before and after the onset of 5-s red light stimulus are plotted in blue and red, respectively. Recorded at 30 fps and rendered at 30 fps.

Title: Supplementary Movie 5

Description: MDNs activate LBL40 neurons. VNC volume of a *VT049484-ZpLexADBD, VT050660-p65ADZp>CsChrimson, VT029570-GAL4>GCaMP6s* fly imaged while MDNs were activated using red light (red squares). Left, maximal-Z projection of raw images averaged across 10 sessions. Right, corresponding ΔF/F_0_ images with color code indicated at the lower right corner. Recorded at 1 fps and rendered at 5 fps.

Title: Supplementary Movie 6

Description: LBL40 activation triggers tibia flexion. A decapitated *SS47938>CsChrimson* fly presented with five 50-ms red light pulses (red squares) at 1-s intervals. All presentations elicit tibia flexion; for the third and the fifth stimuli, flexion is followed by re-extension. Recorded at 200 fps and rendered at 40 fps.

Title: Supplementary Movie 7

Description: MDNs preferentially activate LUL130 in T3. VNC volume of a *VT049484-ZpLexADBD, VT050660-p65ADZp>CsChrimson, VT062245-GAL4>GCaMP6s* fly imaged while MDNs were activated using red light (red squares). Left, maximal-Z projection of raw images averaged across 10 sessions. Right, corresponding ΔF/F_0_ images with color code indicated at the lower right corner. Recorded at 1 fps and rendered at 5 fps.

Title: Supplementary Movie 8

Description: LUL130 activation triggers leg lifting. A decapitated *SS50974>CsChrimson* fly presented with five 5-ms red light pulses (red squares) at 1-s intervals. All presentations elicit hindleg lifting. Recorded at 200 fps and rendered at 40 fps.

Title: Supplementary Movie 9

Description: Coordinated stepping induced by LUL130 activation. A decapitated *SS50975>CsChrimson* fly presented with a 5-s red-light pulse (red square), resulting in coordinated stepping across all 6 legs. Recorded at 200 fps and rendered at 40 fps.

Title: Supplementary Movie 10

Description: Silencing LBL40 neurons during backward walking results in a slower stroke. A decapitated *MDN>CsChrimson, SS47938>GtACR2* fly presented with a 25-s red-light pulse (red square) to activate MDN and trigger backward walking, during which a 10-s blue light pulse (blue square) was presented to silence LBL40 neurons. Flexion of the hindleg femur-tibia joint is noticeably slower during the blue light stimulus. Recorded and rendered at 200 fps.

Title: Supplementary Movie 11

Description: Silencing LUL130 neurons during backward walking delays swing phase. A decapitated *MDN>CsChrimson, SS50974>GtACR2* fly presented with a 25-s red-light pulse (red square) to activate MDN and trigger backward walking, during which a 10-s blue light pulse (blue square) was presented to silence LUL130 neurons. The hindleg femur-tibia joint reaches an abnormally acute angle and swing phase is noticeably delayed during the blue light stimulus. Recorded and rendered at 200 fps.

Title: Supplementary Data

Description: Summary of MF cell types and corresponding fly lines.
