# Supplementary material for: Distributed control of motor circuits for backward walking in Drosophila
Source: Nat Commun. 2020 Dec 2;11:6166. doi: 10.1038/s41467-020-19936-x (PMC7710706; doi:10.1038/s41467-020-19936-x)
Supplement: Supplementary file 15 — Reporting Summary [file 41467_2020_19936_MOESM15_ESM.pdf]

Corresponding author(s): Kai Feng
Barry J. Dickson

Last updated by author(s): Oct 29, 2020

Reporting Summary

Nature Research wishes to improve the reproducibility of the work that we publish. This form provides structure for consistency and transparency in reporting. For further information on Nature Research policies, see our Editorial Policies and the Editorial Policy Checklist.

Statistics

For all statistical analyses, confirm that the following items are present in the figure legend, table legend, main text, or Methods section.

n/a	Confirmed	
☐	☒	The exact sample size (*n*) for each experimental group/condition, given as a discrete number and unit of measurement
☐	☒	A statement on whether measurements were taken from distinct samples or whether the same sample was measured repeatedly
☐	☒	The statistical test(s) used AND whether they are one- or two-sided<br>*Only common tests should be described solely by name; describe more complex techniques in the Methods section.*
☒	☐	A description of all covariates tested
☐	☒	A description of any assumptions or corrections, such as tests of normality and adjustment for multiple comparisons
☐	☒	A full description of the statistical parameters including central tendency (e.g. means) or other basic estimates (e.g. regression coefficient) AND variation (e.g. standard deviation) or associated estimates of uncertainty (e.g. confidence intervals)
☐	☒	For null hypothesis testing, the test statistic (e.g. F, t, r) with confidence intervals, effect sizes, degrees of freedom and P value noted<br>*Give P values as exact values whenever suitable.*
☒	☐	For Bayesian analysis, information on the choice of priors and Markov chain Monte Carlo settings
☒	☐	For hierarchical and complex designs, identification of the appropriate level for tests and full reporting of outcomes
☒	☐	Estimates of effect sizes (e.g. Cohen's *d*, Pearson's *r*), indicating how they were calculated

Our web collection on statistics for biologists contains articles on many of the points above.

Software and code

Policy information about availability of computer code

Data collection	ThorImage 3.1 was used to collect functional imaging data; FLIR Spinnaker SDK 1.23.0.27 (Windows 64bit), Basler Pylon software 6.0.13.7126 (Windows 64bit) and custom MATLAB codes written in MATLAB 2018b were used to collect high speed videos.
Data analysis	DeepLabCut 2.0 was used to analyze joint kinematics; MATLAB implementation of NoRMCorre (https://github.com/flatironinstitute/NoRMCorre) and custom MATLAB codes written in MATLAB 2018b were used to analyze functional imaging and joint kinematics data; imagej 1.52i was used to analyze functional imaging and immunohistochemistry data; VVD viewer 1.0.0 was used to segment neurons from confocal images; GraphPad Prism 8.0 was used to plot figures and perform statistical analysis.

For manuscripts utilizing custom algorithms or software that are central to the research but not yet described in published literature, software must be made available to editors and reviewers. We strongly encourage code deposition in a community repository (e.g. GitHub). See the Nature Research guidelines for submitting code & software for further information.

Data

Policy information about availability of data

All manuscripts must include a data availability statement. This statement should provide the following information, where applicable:
- Accession codes, unique identifiers, or web links for publicly available datasets
- A list of figures that have associated raw data
- A description of any restrictions on data availability

Confocal images of the central nervous systems of split-GAL4 lines used in this study are available at http://splitgal4.janelia.org/cgi-bin/splitgal4.cgi. Other datasets generated during the current study are available from the corresponding author on reasonable request.

Field-specific reporting

Please select the one below that is the best fit for your research. If you are not sure, read the appropriate sections before making your selection.

☒ Life sciences ☐ Behavioural & social sciences ☐ Ecological, evolutionary & environmental sciences

For a reference copy of the document with all sections, see nature.com/documents/nr-reporting-summary-flat.pdf

Life sciences study design

All studies must disclose on these points even when the disclosure is negative.

Sample size	Samples sizes were chosen based on prior experience with similar behavioral and imaging experiments. No sample-size calculation was performed.
Data exclusions	A small fraction of videos for joint kinematics, which were obviously mislabeled by the DeepLabCut model, were excluded.
Replication	Experiments were performed independently for at least three times. All attempts at replication were successful.
Randomization	Animals were allocated into experimental groups based on their genotypes. For the same genotype, flies were randomly picked from the food vials.
Blinding	Experimenters were not blind to genotypes, because our behavioral and calcium imaging data analysis was non-subjective.

Reporting for specific materials, systems and methods

We require information from authors about some types of materials, experimental systems and methods used in many studies. Here, indicate whether each material, system or method listed is relevant to your study. If you are not sure if a list item applies to your research, read the appropriate section before selecting a response.

Materials & experimental systems

n/a	Involved in the study	
☐	☒	Antibodies
☒	☐	Eukaryotic cell lines
☒	☐	Palaeontology and archaeology
☐	☒	Animals and other organisms
☒	☐	Human research participants
☒	☐	Clinical data
☒	☐	Dual use research of concern

Methods

n/a	Involved in the study	
☒	☐	ChIP-seq
☒	☐	Flow cytometry
☒	☐	MRI-based neuroimaging

Antibodies

Antibodies used	Rabbit polyclonal anti-GFP (Merck/Millipore, AB-3080P); Rabbit polyclonal anti-GFP (Thermo Fisher Scientific, A11122); Rat monoclonal anti-HA (Roche, 11867423001); Mouse monoclonal anti-Bruchpilot (Developmental Studies Hybridoma Bank, Cat# nc82 s; RRID:AB_2314866); Rat monoclonal anti-RFP 5F8 (Chromotek, 5f8-20); Rabbit polyclonal POL 016 anti-Tetanus Toxin (Statens Serum Institute, 65873); Rat monoclonal anti-FLAG Tag (Novus Biologicals, NBP1-06712); Rabbit monoclonal anti-HA Tag (Cell Signal Technologies, 3724S); DL550 Mouse monoclonal anti-V5 (AbD Serotec, MCA1360D550GA); Cy2 SNAP-tag ligand (Luke Lavis, JRC, N/A) ATTO647N Goat anti-Rat (Rockland, 612-156-120); AF594 Donkey anti-Rabbit (Jackson ImmunoResearch, 711-585-152); Cy5 goat anti-mouse (Jackson ImmunoResearch, 115-175-166); Cy3 goat anti-rat (Jackson ImmunoResearch, 112-165-167); Alexa Fluor 555 goat anti-rat (Thermo Fisher Scientific, A-21434); Alexa Fluor 488 goat anti-rabbit (Thermo Fisher Scientific, A-11034); Alexa Fluor 568 goat anti-mouse (Thermo Fisher Scientific, A-11031); Alexa Fluor 647 goat anti-mouse (Thermo Fisher Scientific, A-21236); Alexa Fluor 647 goat anti-rabbit (Thermo Fisher Scientific, A-21245).
Validation	All antibodies were commonly-used commercial antibodies that were validated by the manufacturer and used in previous publications, in various species including flies, except for the Cy2 SNAP-tag ligand, which were validated and used by the Janelia Flylight team routinely, see Meissner et al., 2020. See below for links containing the validation information and references for all primary antibodies:<br>https://www.emdmillipore.com/US/en/product/Anti-Green-Fluorescent-Protein-Antibody,MM_NF-AB3080P<br>https://www.thermofisher.com/antibody/product/GFP-Antibody-Polyclonal/A-11122<br>https://www.sigmaaldrich.com/catalog/product/roche/roahaha?lang=en&region=AU<br>https://dshb.biology.uiowa.edu/nc82<br>https://www.chromotek.com/products/detail/product-detail/rfp-antibody-5f8/<br>https://antibodies.ssi.dk/-/media/arkiv/subsites/antibodies/product-specifications/product-specification---pol-016-anti-tetanus-toxin-tent-65873.pdf<br>https://www.novusbio.com/products/dykddddk-epitope-tag-antibody-l5_nbp1-06712

https://www.cellsignal.com/products/primary-antibodies/ha-tag-c29f4-rabbit-mab/3724?Ntk=Products&Ntt=3724
https://www.labome.com/product/Bio-Rad/MCA1360D550GA.html

Animals and other organisms

Policy information about studies involving animals; ARRIVE guidelines recommended for reporting animal research

Laboratory animals	Transgenic Drosophila melanogaster strains used in this study. For all behavioral experiments and immunostainings, 3-8 day old flies of both sexes were used, except for MDN amputation and GtACR neuronal epistasis experiments, in which only females were used. For trans-Tango experiments, 15-40 day old flies of both sexes were used. For calcium imaging, 3-20 day old females were used.
Wild animals	No wild animals were used.
Field-collected samples	This study did not involve samples collected from the field.
Ethics oversight	No ethical approval was required because only insects were used in this study

Note that full information on the approval of the study protocol must also be provided in the manuscript.
